# Astrocytes acquire resistance to iron-dependent oxidative stress upon proinflammatory activation

**DOI:** 10.1186/1742-2094-10-130

**Published:** 2013-10-28

**Authors:** Romina Macco, Ilaria Pelizzoni, Alessandra Consonni, Ilaria Vitali, Giacomo Giacalone, Filippo Martinelli Boneschi, Franca Codazzi, Fabio Grohovaz, Daniele Zacchetti

**Affiliations:** 1 Division of Neuroscience, Dibit, San Raffaele Scientific Institute, via Olgettina 58, 20132, Milano, Italy; 2 Vita-Salute San Raffaele University, via Olgettina 58, 20132, Milano, Italy

**Keywords:** Astrocyte activation, Oxidative stress, Iron, Cytokines, Nrf2, Microglia

## Abstract

**Background:**

Astrocytes respond to local insults within the brain and the spinal cord with important changes in their phenotype. This process, overall known as “activation”, is observed upon proinflammatory stimulation and leads astrocytes to acquire either a detrimental phenotype, thereby contributing to the neurodegenerative process, or a protective phenotype, thus supporting neuronal survival. Within the mechanisms responsible for inflammatory neurodegeneration, oxidative stress plays a major role and has recently been recognized to be heavily influenced by changes in cytosolic iron levels. In this work, we investigated how activation affects the competence of astrocytes to handle iron overload and the ensuing oxidative stress.

**Methods:**

Cultures of pure cortical astrocytes were preincubated with proinflammatory cytokines (interleukin-1β and tumor necrosis factor α) or conditioned medium from lipopolysaccharide-activated microglia to promote activation and then exposed to a protocol of iron overload.

**Results:**

We demonstrate that activated astrocytes display an efficient protection against iron-mediated oxidative stress and cell death. Based on this evidence, we performed a comprehensive biochemical and molecular analysis, including a transcriptomic approach, to identify the molecular basis of this resistance.

**Conclusions:**

We propose the protective phenotype acquired after activation not to involve the most common astrocytic antioxidant pathway, based on the Nrf2 transcription factor, but to result from a complex change in the expression and activity of several genes involved in the control of cellular redox state.

## Background

Astrocytes represent the most abundant cell type in the central nervous system (CNS), and they are key players for both physiological neuronal functions and pathological processes. They have long been recognized to provide energetic, metabolic and structural support to neurons through the secretion of nutrients and the maintenance of the blood–brain barrier integrity [1]. More recently, they have been shown to exert a critical role in the extracellular milieu homeostasis by controlling the concentration of ions, mediators and neurotransmitters, thereby contributing to bidirectional neuron-glia communication [2].

Astrocytes are capable of undergoing rapid changes in their phenotype as a result of alterations of CNS homeostasis. This process, known as “astrocyte activation” or astrogliosis, is a well-established feature of several pathological situations [3], in particular within neuroinflammatory processes, in which an intense glial fibrillary acidic protein staining can be observed [4,5]. The way astrocyte activation contributes to an either neurodegenerative or neuroprotective role is still an open question [6].

In this scenario, one important role that astrocytes might play is related to oxidative stress, a condition that has been ascribed to the reduction of antioxidant defenses and/or to the increase in reactive oxygen species (ROS) production [7]. Indeed, neurons are considered particularly vulnerable to oxidative stress because of their high oxygen rate [8], and oxidative stress has been associated with neurodegeneration during aging, as well as in several disorders, including Parkinson’s and Alzheimer’s diseases [9]. Glial cell activation might also occur in response to oxidative stress and involves a complex range of responses that can either accelerate or delay the neurotoxic effects [10]. Within this framework, growing attention has been paid to the iron content, since it is known to favor free radical generation by reacting with the H_2_O_2_ physiologically produced by the cellular metabolism [11,12].

Several therapeutic strategies addressing oxidative stress toxicity (e.g., the exogenous antioxidant supply) and iron accumulation (e.g., metal chelation therapy) have been attempted, and some proved to be effective (see, for instance, [9,11,12]). While molecules with a direct protective effect on neurons have been searched for, less attention has been paid to astrocytes, even though they are an important alternative target, being a cellular link between oxidative stress toxicity and neuroinflammation [13], as well as key players in non-cell autonomous mechanisms of neurodegeneration [14]. Moreover, astrocytic activity is reported to strongly condition iron homeostasis in the CNS [15]. In fact, they control iron flow through the blood–brain barrier and iron distribution to neurons. Since most of the extracellular iron in the CNS is not bound to transferrin, astrocytes also play an important role in buffering local iron changes, thereby preventing oxidative stress in neurons (see [16] and references therein).

In order to investigate the molecular basis and the effects of the astrocytic activation process under conditions of oxidative stress, proper cellular models and reliable in vitro assays are required. Many studies exploit the use of continuous cell lines that, although easier to handle, substantially differ from primary cultures in terms of resistance to oxidative stress [17]. Primary glial cultures are a better option, although proper care must be taken in order to reproduce the process of activation that occurs in vivo. In fact, the presence of a heterogeneous mixture of cell types in the culture may strongly affect the outcome of the experiments, especially as regards the contribution of microglia to inflammatory conditions [18,19]. Secondary cultures of pure astrocytes are often required to keep experimental variables under control [18].

In this work, we set up a cellular assay in order to follow iron-induced cell stress in highly standardized pure secondary cultures of astrocytes obtained from rat cortex. From this standpoint, we investigated the molecular aspects that determine the resistance of astrocytes to oxidative stress with particular regards to the effects of proinflammatory activation. We also performed a transcriptomic analysis in order to identify the candidate genes involved in the mechanisms of protection.

## Methods

### Materials

Cell culture media and reagents were from BioWhittaker-Lonza (Basel, Switzerland). Other chemicals, if not otherwise stated, were from Sigma-Aldrich (St Louis, MO, USA). Culture flasks and multiwell plates were from Nalge Nunc (Rochester, NY, USA). Petri dishes were from Falcon BD (Franklin Lakes, NJ, USA).

### Cell culture

The animal use procedures were approved by the Institutional Animal Care and Use Committee of the San Raffaele Scientific Institute. Primary cultures of cortical astrocytes were prepared from 2- to 3-day-old Sprague–Dawley rats (Charles River Italia, Calco, LC, Italy) according to [19]. Briefly, after dissection, cortices were cut into small sections with a razor blade. The pieces were collected and washed twice in Hank’s Balanced Salt Solution supplemented with 10 mM Hepes/Na pH 7.4, 12 mM MgSO_4_, 50 U/ml penicillin and 50 μg/ml streptomycin (Gibco, Grand Island, NY, USA). The tissue was then incubated, in two subsequent steps, with 2.5 mg/ml trypsin type IX in the presence of 1 mg/ml deoxyribonuclease (Calbiochem, La Jolla, CA, USA) for 10 min at 37°C and mechanically dissociated. The supernatant obtained was diluted 1:1 in medium containing 10% donor horse serum (PAA Laboratories GmbH, Pasching, Austria). After centrifugation (100 g for 10 min), cells were plated in Minimum Essential Eagle Medium supplemented with 10% donor horse serum, 33 mM glucose, 2 mM glutamax (Gibco), 50 U/ml penicillin and 50 μg/ml streptomycin. Cells were maintained in 75 cm^2^ flasks (about 1 per pup) at 37°C in a 5% CO_2_ humidified incubator. In order to remove microglia and oligodendrocyte progenitors and obtain pure secondary cultures of astrocytes (>99.8%), flasks were shaken at 200 rpm for 24 h at 37°C at days 2 and 6 after dissection in Minimum Essential Medium with Hank's salts supplemented with 10% donor horse serum, 33 mM glucose, 2 mM glutamax and 10 mM Hepes/Na, pH 7.4. Contamination with non-astrocytic cells was assessed by immunocytochemistry (GFAP for astrocytes, IBA1 for microglia and A2B5 for oligodendrocyte precursors) according to [20]. After reaching confluence, astrocytes were trypsinized and replated in Minimum Essential Medium Eagle supplemented with 10% donor horse serum (BioWhittaker-Lonza or, in comparative experiments, Invitrogen, Carlsbad, CA, USA), 33 mM glucose, 2 mM glutamax, 50 U/ml penicillin and 50 μg/ml streptomycin onto polylysine-coated glass coverslips or plastic multiwells. Cultures were maintained at 37°C in a 5% CO_2_ humidified incubator, and experiments were performed within 3 days after re-plating.

### Dye loading

Dye loading was performed in Krebs-Ringer-Hepes buffer (5 mM KCl, 125 mM NaCl, 2 mM CaCl_2_, 1.2 mM MgSO_4_, 1.2 mM KH_2_PO_4_, 6 mM glucose and 20 mM Hepes, pH 7.4). The fluorescent dyes (from Molecular Probes, Invitrogen, when not specified) were administered as follows: (1) fura-2 acetoxymethyl ester (Calbiochem), 4 μM, 40 min at 37°C; (2) Sytox Blue, 5 μM, kept in the bath during the experiments; (3) 5-(and-6)-chloromethyl-2’,7’-dichlorodihydrofluorescein diacetate, acetyl ester (CM-H2DCFDA), 0.25 μM, 30 min at 37°C; (4) tetramethyl rhodamine methyl ester (TMRM), 25 nM, 15 min at room temperature and maintained in the bath during the experiments. After dye loading, cells were washed once with fresh Krebs-Ringer-Hepes and analyzed in the same buffer. Both single cell and monolayer culture experiments were performed at room temperature.

### Videomicroscopy setup

The videoimaging setup for single cell studies is based on an Axioskope 2 microscope (Zeiss, Oberkochen, Germany) and a Polychrome IV (Till Photonics GmbH, Martinsried, Germany) light source. In the epifluorescence path, fura-2 was excited at 355 nm to monitor Fe^2+^ variations (as quenching of the fluorescence signal). The excitation wavelength of 355 nm was adopted instead of the theoretical 360-nm isosbestic wavelength because it turned out to be insensitive to Ca^2+^ variations in our optical configuration. Fluorescence images were collected by a cooled CCD videocamera (PCO Computer Optics GmbH, Kelheim, Germany). The Vision software (Till Photonics) was used to control the acquisition protocol and to perform data analysis.

### Fluorescence plate reader measurements

The measurements of the fluorescence intensity for pure astrocytic monolayers were based on a plate reader (Mithras LB 940, Berthold Technologies or Wallac Victor3TM 1420 multilabel counter, Perkin Elmer) provided with a flash lamp and sensitive PMTs coupled with high transmission band pass filters. Briefly, treated or untreated astrocytes plated on 24-well plates were loaded with fura-2 or CM-H2DCFDA, as previously described. Fluorescence was measured every 60 s up to 90 min.

### Cellular treatments

Fe^2+^ was prepared as a solution of ferrous-ammonium sulfate, freshly dissolved in water and kept in ice until use. Acute iron overload was performed by incubating fura2-loaded astrocytes with 20 μM pyrithione, an iron ionophore, and Fe^2+^ 1 μM for 3 min to allow iron entry (monitored by fura2 quenching). Cells were then washed twice with Krebs-Ringer-Hepes solution to remove extracellular Fe^2+^, and the fluorescence signal was monitored over time.

In some experiments, a pre-treatment with drugs or pharmacologic agents was needed: (1) L-buthionine-sulfoximine (BSO), an inhibitor of glutathione (GSH) synthesis, 1 mM was added to the cellular medium 24 h before the experiment; (2) Mito-TEMPO [(2-(2,2,6,6-tetramethylpiperidin-1-oxyl-4-ylamino)-2-oxoethyl) triphenylphosphonium chloride monohydrate; ENZO Life Science, Farmingdale, NY, USA] 200 μM, as well as Trolox (6-hydroxy-2,5,7,8-tetramethylchroman-2-carboxylic acid) 1 mM were added to cells 2 h before the experiment; (3) the inhibitor of GSH reductase, 2AAPA (R,R’-2-acetylamino-3-[4-(2-acetylamino-2-carboxyethyl-sulfanylthiocarbonylamino)-phenylthiocarbamoylsulfanyl] propionic acid hydrate S,S’-[1,4-Phenylenebis(iminocarbonothioyl)]bis[N-acetyl-L-cysteine], 25 μM, was administered at the beginning of the experiment (after acute iron overload) and left until the end.

Astrocyte activation was induced by treating cells with recombinant rat interleukin-1β (IL-1β) and tumor necrosis factor α (TNFα) from R&D Systems (Minneapolis, MN, USA). Stimuli were administered directly to the culture medium 24 h before the experiment as follows: 10 ng/ml IL-1β and 30 ng/ml TNFα. Lipopolysaccharide (LPS) (10 ng/ml) was added directly to the astrocyte culture medium 24 h before the experiment and used as a negative control for activation. Conditioned medium obtained from resting or activated (with 10 ng/ml LPS) microglia was incubated on astrocytes in substitution for their culture medium 24 h before the experiment.

### GSH measurement

Reduced glutathione in pure astrocytic cultures was measured by using QuantiChromTM Glutathione Assay Kit (DIGIT-250) from BioAssay Systems (Hayward, CA, USA). Treated or untreated astrocytes were washed and then lysed for 15 min at 4°C with lysis buffer (150 μl for two 35 × 10 petri dishes) containing phosphate-buffered saline (137 mM NaCl, 2.7 mM KCl, 10 mM sodium phosphate dibasic, 2 mM potassium phosphate monobasic, pH 7.4) supplemented with 2% Nonidet P-40, 10 mM EDTA/Na and a cocktail of protease inhibitors (chymostatin, leupeptin, antipain and pepstatin dissolved 1000x in dimethylsulfoxide and used 10 μg/ml each). Lysates were centrifuged for 15 min at 15,000 g at 4°C, and 120 μl of the supernatant was mixed with 120 μl of provided Reagent A (for deproteination and detection). After vortexing, the sample/reagent A mixture was centrifuged for 5 min at 15,000 g. Eventually, 200 μl of the supernatant was added with 100 μl of provided reagent B, i.e., 5,5’-dithiobis-2-nitrobenzoic acid, for colorimetric reaction and transferred into the wells of a 96-well plate. After an incubation of 25 min at room temperature, absorbance of the samples was read at 412 nm. To confirm the obtained data, we also employed a Glutathione Fluorometric Assay Kit from Biovision (Milpitas, CA, USA) according to the manufacturer’s instructions.

### Cell viability assay

Cell viability was quantified by 3-(4,5-dimethylthiazol-2-yl)-2,5-diphenyltetrazolium bromide (MTT) assay. Briefly, after treatment, pure cultures of astrocytes plated on 24-well plates were incubated for 1 h at 37°C with 0.5 mg/ml MTT in culture medium. After removing the extracellular solution, formazan, the MTT metabolic product, was dissolved in dimethylsulfoxide, and the absorbance was read at 570 nm.

### DNA and siRNA transfection

DNA and siRNA transfections were performed using Lipofectamine 2000 reagent (Life Technologies). Briefly, DNA or siRNA (1.8 μg/ml and 200 pmol/ml, respectively), as well as lipofectamine 2000 (4 μl/ml), was separately incubated with Optimem Reduced Serum Media (Gibco) for 5 min at room temperature. Subsequently, the Lipofectamine mix was added to DNA or siRNA mix and left for 30 min at room temperature. Astrocytes plated on 35 × 10-mm petri dishes or 24 multiwell plates were washed once with Optimem and incubated with the transfection solution for 90 min at 37°C. After two washes with Optimem, astrocyte-conditioned medium was eventually put on the cells. Twenty-four hours of expression was required before performing the experiments. SiRNAs used (forward and reverse) were: (1) cgaccuacgugaacaaucutt and agauuguucacguaggucgcg for the gene encoding mitochondrial superoxide dismutase (SOD2); (2) agcuaguguagaaauaauatt and uauuauuucuacacuagcutt plus cgaguuacagugucuuaautt and auuaagacacuguaacucggg for the nuclear factor (erythroid-derived 2)-like 2 gene (NFE2L2) encoding Nrf2.

### Western blotting

Cells were washed twice with phosphate-buffered saline and lysed for 15 min at 4°C with 100 μl/petri dish (35 × 10 mm) of lysis buffer (phosphate-buffered saline supplemented with 2% Nonidet P-40, 0.2% sodium dodecyl sulfate, 10 mM EDTA/Na and the cocktail of protease inhibitors).

Lysates were centrifuged for 15 min at 15,000 g at 4°C, and the supernatants were collected and their total protein content analyzed by the MicroBCA reagent (ThermoFisher Scientific, Pierce, Waltham, MA, USA). About 20–40 μg of proteins was separated by standard sodium dodecyl sulfate polyacrylamide gel electrophoresis and then electrically transferred onto a nitrocellulose membrane in blotting buffer [2.5 mM tris(hydroxymethyl)aminomethane, 19.2 mM glycine, 20% methanol]. The nitrocellulose filter was stained with Ponceau S (0.2% in 3% trichloroacetic acid) and de-stained with double-distilled water for protein visualization.

Typically, membranes were blocked in washing solution (10 mM tris(hydroxymethyl)aminomethane/HCl, 150 mM NaCl, 0.1% Tween-20, pH 7.6) containing 5% non-fat dry milk overnight at 4°C. Primary antibodies were diluted in blocking solution and incubated for 2 h at room temperature. Subsequently, membranes were washed three times for 5 min with washing solution and incubated 1 h with goat anti-rabbit or anti-mouse horseradish peroxidase-conjugated secondary antibody (ThermoFisher Scientific, Pierce) diluted in blocking solution at room temperature. After three washing steps, protein bands were detected on auto-radiographic films (GE Healthcare, Piscataway, NJ, USA) by incubation with chemiluminescent solutions (Pico or Super Signal West Femto chemiluminescent kit, ThermoFisher Scientific, Pierce). The following primary antibodies were used: (1) rabbit polyclonal anti-SOD2 antibody from Merck Millipore (Billerica, MA, USA); (2) mouse monoclonal anti-catalase antibody from Sigma Aldrich; (3) rabbit polyclonal anti-peroxiredoxin I, III and V antibodies from AbFrontier (Korea); (4) goat polyclonal anti-peroxiredoxin VI antibody from R&D Systems; (5) rabbit polyclonal anti-SOD1 antibody from Santa Cruz Biotechnology (Santa Cruz, CA, USA).

### RNA extraction, reverse transcription and quantitative PCR

RNA was extracted from treated or untreated cells plated on 35 × 10-mm Petri dishes with TRIzol (Invitrogen) and phenol/chlorophorm/isoamyl alcohol (25:24:1 v/v), following the manufacturer’s instruction. Briefly, cells were lysed in 1 ml of TRIzol, to which 200 μl of phenol/chlorophorm/isoamyl alcohol was added. After centrifugation (12,000 g, 15 min.), the upper aqueous phase was transferred in a new tube, and RNA was precipitated through addition of an equivalent amount of isopropanol. Samples were centrifuged (12,000 g, 10 min) and washed with 70% ethanol. RNA pellets were air-dried for 5 min, resuspended in 20 μl of RNase-free water and stored at −80°C.

Reverse transcription (RT) was carried out with random hexamers as primers, using the Superscript III Retrotranscription Kit (Invitrogen) following the manufacturer’s instructions. RT was performed at 50°C for 50 min, then incubating samples were stopped at 85°C for 5 min. Single-strand cDNA was obtained by digesting complementary RNA strands with provided RNase H for 20 min at 37°C.

Quantitative polymerase chain reaction (qPCR) was performed on a LightCycler 480 machine (Roche Diagnostics, Basel, Switzerland), with proprietary SybrGreen mix (LightCycler 480 Master Mix, Roche), following the manufacturer’s instructions. Both forward and reverse primers were used at a 0.5 μM concentration. RT-derived cDNA was typically diluted 1:4 before use. A PCR program was performed with 10 min of the denaturation step at 95°C and 35 to 45 cycles of amplification. Each cycle consisted of a denaturation step (95°C, 10 s), annealing step (60°C, 25 s) and elongation step (72°C, 15 s). After amplification, a melting step was performed (95°C for 30 s, 60°C for 1 min). Determination of crossing points and melting peaks was performed with LightCycler 480 Software (version 1.5.0.39, Roche). Primers used (forward and reverse) were: (1) gacctacgtgaacaatctgaacg and cttgatagcctccagcaactct for the gene encoding SOD2; (2) gcagagacattcccatttgtagat and cttaaatcagtcatggccgtct for the gene encoding Nrf2; (3) tcaccattaagctgggcg and ttcttcccggtccagtcata for the gene encoding frataxin; (4) gtatgaacagcgatgatgcact and gaagaccagagcagattttcaatag for the gene encoding interleukin-6 (used as a positive control for activation) and (5) gaagaagaaattagagaagcgttcc and gtagtttacctgaccatccccat for calmodulin 2 (used as an internal reference for normalization).

### Transcriptomic analysis

After the treatments, astrocytes were subjected to RNA extraction using Trizol (Invitrogen), according to the manufacturer’s instructions. The gene expression profiling was determined using RatRef-12 Expression Beadchips (Illumina Inc., San Diego, CA, USA). Each beadchip investigates for more than 21,000 transcripts selected primarily from the NCBI RefSeq database (Release 16) and in a minor part from the UniGene database. An amount of 500 ng of total RNA was reverse transcribed into complementary RNA and biotin-UTP labeled using the Illumina TotalPrep RNA Amplification Kit (Applied Biosystems) according to the manufacturer’s protocol; 750 ng of complementary RNA was then hybridized to the BeadChip array and stained with streptavidin-Cy3. All procedures were performed following the manufacturer’s instructions. BeadChips were imaged using the Illumina BeadArray Reader, a two-channel 0.8-μm-resolution confocal laser scanner and Illumina BeadScan software. Illumina GenomeStudio v.2011.1 software was used to elaborate the fluorescence signal to a value whose intensity corresponded to the quantity of the respective transcript in the original sample. The same software was used to assess the quality controls, which included the biological specimen controls, hybridization controls, signal generation controls and negative controls. The samples belonging to three different experimental conditions (i.e., resting astrocytes, astrocytes stimulated with cytokines and astrocytes stimulated with microglia-conditioned medium) were tested in technical duplicates, and a scatter plot with a correlation coefficient for each couple of replicates was calculated, leading to a mean value of 0.99. The fold change based on raw data was calculated for the comparisons.

Data analysis was performed using the Ingenuity Pathway Analysis (IPA) software (Ingenuity Systems Inc., Redwood City, CA, USA). lllumina probes, filtered for *p* < 0.05 and a threshold of 1.5 points of fold change between different conditions (in upregulation or downregulation) were uploaded into the software together with their differential expression *p*-values measured by *t*-test. Each probe was mapped to its own gene object in the Ingenuity Pathways Knowledge Database.

To interpret the gene expression results of the different condition of stimulation in the context of biological processes, pathways and networks, IPA Core Analyses were conducted. Networks of these genes were assigned a score based on their connectivity: the score reflected the number of focus genes in the network and how relevant this network is to the original list of focus genes. The significance of the association between the data set of genes and the canonical pathways contained in the Ingenuity Pathways Knowledge Database was determined by adjusted *p*-values using the Benjamini-Hochberg correction for multiple testing, and a *p*-value < 0.05 after correction was considered statistically significant according to functional enrichment analysis. Besides, the “overlay” function was used to give a graphic visualization of which subset of genes inside a given canonical pathway was modulated in different conditions.

### Data analysis

Data are presented as mean ± SEM of at least three independent experiments. Statistical significance was tested by using either paired or unpaired two-tailed Student’s *t*-test or one-way ANOVA followed by the Bonferroni (for all pairwise comparisons) post hoc test. Statistical analysis was performed using GraphPad Prism (GraphPad Software, San Diego, CA, USA).

## Results

### Iron-dependent oxidative stress in cortical astrocytes

**Figure 1:**
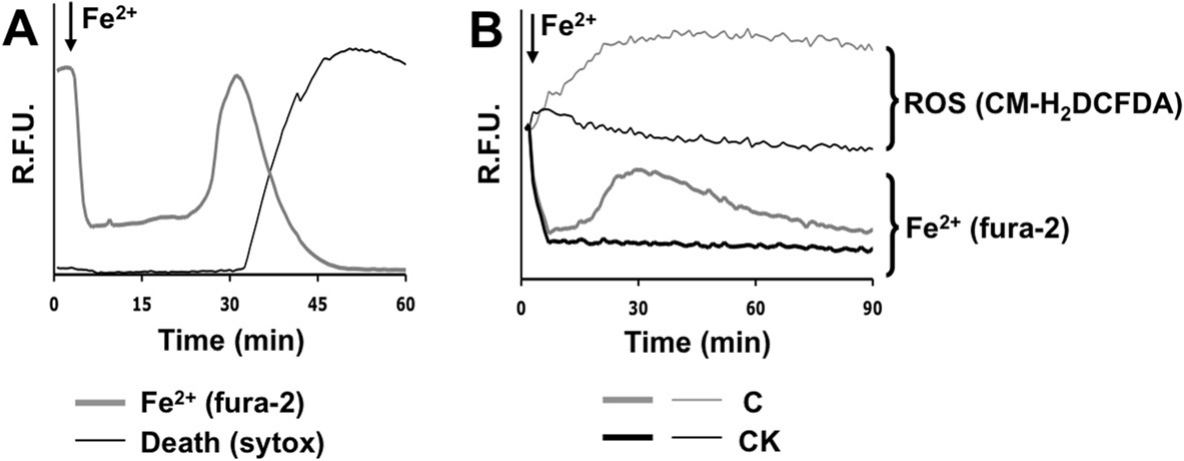
**Activation protects astrocytes from iron-dependent oxidative stress and cell death. (A)** Acute 1 μM iron overload (from this graph onwards indicated as Fe^2+^) was performed, and, after washing away extracellular Fe^2+^, the fluorescence of fura-2 (iron oxidative status) and Sytox Blue (cell death) were monitored for up to 60 min. The temporal analysis traces show the behavior of a single representative astrocyte in a microscopic field (expressed as relative fluorescence units, RFU). In this and in the following figures, the graphs are illustrative of at least three independent experiments. **(B)** A monolayer of pure astrocytes was left untreated (C) or activated for 24 h with a mix of two proinflammatory cytokines (CK: 10 ng/ml IL1β and 30 ng/ml TNFα). Cells were loaded with fura-2 (iron oxidative status) or CM-H2DCFDA (ROS detection) and then challenged with the acute iron overload. The traces represent the fluorescence intensity (expressed as RFU) of the astrocytes plated in a well of a 24-well plate (about 250,000 cells).

In order to evaluate the effects of oxidative stress on pure cortical astrocytes in culture, we exposed cells to 1 μM Fe^2+^ (administered as ferrous ammonium sulfate) for a short time in the presence of 20 μM pyrithione, an iron ionophore. Afterwards, cells were washed to stop iron entry, and fluorescence was followed over time. This iron overload protocol allows fast iron entry and a controlled increase in its cytosolic concentration [21]. The intracellular levels of Fe^2+^ concentration were monitored by single-cell videomicroscopy, by exploiting the capability of Fe^2+^ to quench fura-2 fluorescence (excitation at 360 nm, i.e., at the calcium insensitive wavelength). This dye was preferred to calcein, a widely used iron probe [22], since its affinity, higher for Fe^2+^ (Kd 10^-9^) than for Fe^3+^ (Kd 10^-6^), allows the discrimination between the two iron forms (see also [17]). Quenching of intracellular fura-2 (i.e., the readout for Fe^2+^ entry) was first observed when the extracellular concentration of Fe^2+^ was raised above 0.3 μM—thereby suggesting a basal level of free intracellular Fe^2+^ around 0.5 μM—and was ~50% in the presence of 1 μM (data not shown). The fluorescence quenching caused by Fe^2+^ entry was maintained for a variable period of time, after which a rapid and complete recovery of the signal was observed (Figure 1A, gray thick trace). This dequenching phase was most likely due to the oxidation of Fe^2+^ to Fe^3+^ during the self-regenerative Fenton reaction. In fact, it could also be triggered by administration of H_2_O_2_ (data not shown; see also [17]). After a few seconds, the dequenching was followed by a loss of plasma membrane integrity, as indicated by both the drop of the fura-2 fluorescence signal (Figure 1A, gray thick trace) and the concomitant nuclear staining by Sytox blue, a fluorescent cell death indicator (Figure 1A, black thin trace).

Similar kinetics were observed when the iron overload was investigated on a cell monolayer (analyzed by a plate reader), even though fluorescence variations were smoother, reflecting the integration of the asynchronous signals coming from individual cells (Figure 1B, gray thick trace). Accordingly, all following experiments were performed on astrocyte monolayers if not otherwise specified. In order to evaluate the effects of iron overload on the intracellular production of ROS, astrocyte monolayers were also loaded with CM-H2DCFDA, a reduced derivative of fluorescein, whose oxidation by ROS induces an increase in fluorescence signal. ROS accumulation was observed to precede the rise of fura-2 fluorescence (Figure 1B, gray thin trace).

Since recent evidence suggested a protective role of astrocyte activation against the oxidative stress induced by H_2_O_2_[23], we investigated whether this was the case also in a condition of iron-mediated oxidative stress. Astrocytes were treated in vitro for 24 h with two proinflammatory cytokines (CK: 10 ng/ml IL-1β + 30 ng/ml TNFα), and the changes in their phenotype were assessed by various activation markers, including interleukin-6, NO and inducible NO synthase (data not shown, see also [19]). When cytokine-activated astrocytes were exposed to the iron overload protocol, after the initial quenching, fura-2 fluorescence remained stable, with signs of neither iron oxidation nor membrane alterations (Figure 1B, black thick trace). Moreover, no ROS increase was observed (Figure 1B, black thin trace). These results suggest that, in astrocytes, ROS accumulation is responsible for cell death after iron overload and activation confers resistance.

### Effects of iron overload on mitochondria

**Figure 2:**
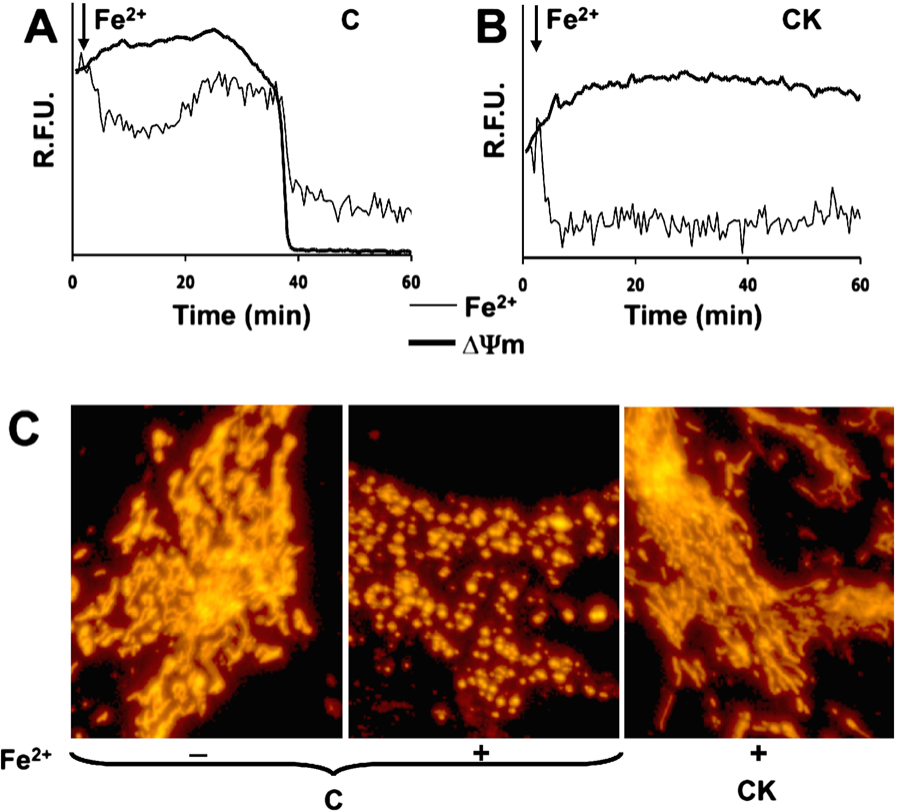
**Activated astrocytes are protected from mitochondrial damage induced by acute iron overload. (A, B)** Cells were loaded with both fura-2 and TMRM in order to monitor the iron oxidative status (Fe^2+^) and mitochondrial membrane potential (ΔΨm), respectively. After iron overload, the resting astrocyte **(A)** showed a progressive ΔΨm decay before cell death (i.e., the drop of fura-2 signal). The signal of both fura-2 and TMRM, on the contrary, remained stable in activated astrocytes (CK) challenged with iron overload **(B)**. The traces represent the temporal analysis of the fluorescence within a single representative astrocyte in a microscopic field (expressed as RFU). **(C)** In order to evaluate mitochondrial morphology, cells were transfected with a mitochondrial red fluorescent protein (left panel). Iron overload had a strong effect on the mitochondrial morphology in resting astrocytes (middle panel). In contrast, mitochondrial shape was preserved in activated astrocytes (right panel).

Since mitochondria represent the main site of iron utilization and ROS production, we investigated the effects of iron overload on both their membrane potential and morphology, looking at differences between resting and activated astrocytes. Astrocytes were loaded with both fura-2 and TMRM, a probe for mitochondrial membrane potential, exposed to iron and analyzed at the single cell level. In astrocytes at rest, TMRM fluorescence showed a fast drop that just preceded the loss of fura-2 fluorescence, i.e., the sign of cell death (Figure 2A), thereby suggesting an impairment of mitochondrial functionality in the oxidative stress-mediated cell death. When astrocytes were activated by 24 h pretreatment with IL-1β and TNFα, the mitochondrial membrane potential was preserved, and the cells were protected (Figure 2B). Of note, iron overload also led to alteration of the mitochondrial morphology, converting the mitochondrial shape (made visible by overexpression of a fluorescent protein targeted to mitochondria) from elongated to fragmented (Figure 2C, compare left panel with middle panel). The activation process plays a protective role, preserving mitochondrial morphology after iron challenge (Figure 2C, right panel).

### Effect of microglia activation on the astrocytic phenotype

**Figure 3:**
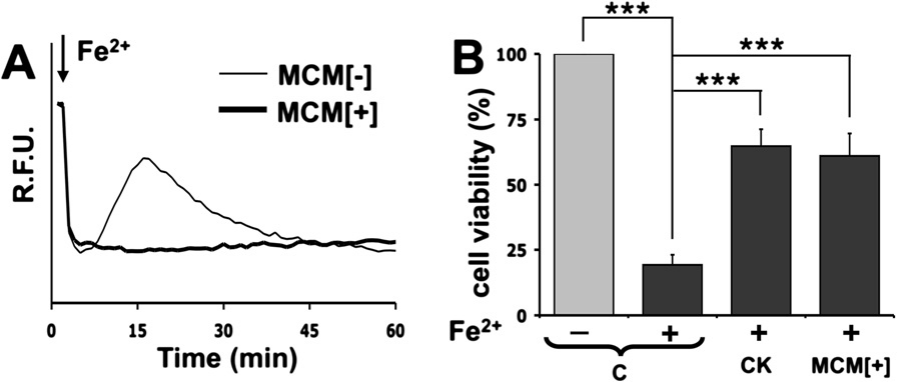
**Conditioned medium from LPS-activated microglia protects astrocytes from iron-mediated toxicity. (A)** A monolayer of astrocytes was grown for 24 h with conditioned medium from either quiescent microglia (MCM[−]) or 10 ng/ml LPS-activated microglia (MCM[+]). Following acute iron overload, astrocytes, treated with MCM[−], underwent iron oxidation and membrane damage detected by fura-2 variations. MCM[+]-treated astrocytes showed resistance. The traces represent the fluorescence intensity analyzed with a plate reader and expressed as RFU. **(B)** Pure cultures of astrocytes were left untreated (C) or treated for 24 h with either cytokines (CK) or MCM[+], and analyzed 90 min after acute iron overload. Cell viability was measured by MTT assay. Statistical significance (***p < 0.001) was calculated using one-way ANOVA followed by Bonferroni post hoc test.

Since the pretreatment with cytokines might not be representative of the complex interplay between activated microglia and astrocytes, we employed a different protocol of activation [23] that is expected to better mimic the in vivo condition. Astrocytes were treated for 24 h with conditioned medium obtained from quiescent (control) or LPS-treated microglia (MCM[−] and MCM[+], respectively). Also in this experiment, astrocytes activated by pre-incubation with MCM[+] were protected from the toxic effects of iron and did not show the typical rise of fura-2 fluorescence (oxidative stress) with ensuing cell death (Figure 3A). This result was not influenced by the presence of LPS in the medium since direct administration of LPS to our astrocyte cultures (virtually devoid of contaminating microglia; see Methods and [19]) induced neither activation nor protection from oxidative stress (data not shown). The protecting phenotype obtained by incubation of astrocytes with either the two pro-inflammatory cytokines or the MCM[+] was further confirmed by an analysis of cell viability performed with the colorimetric MTT assay (Figure 3B).

### Role of glutathione and ROS scavengers

**Figure 4:**
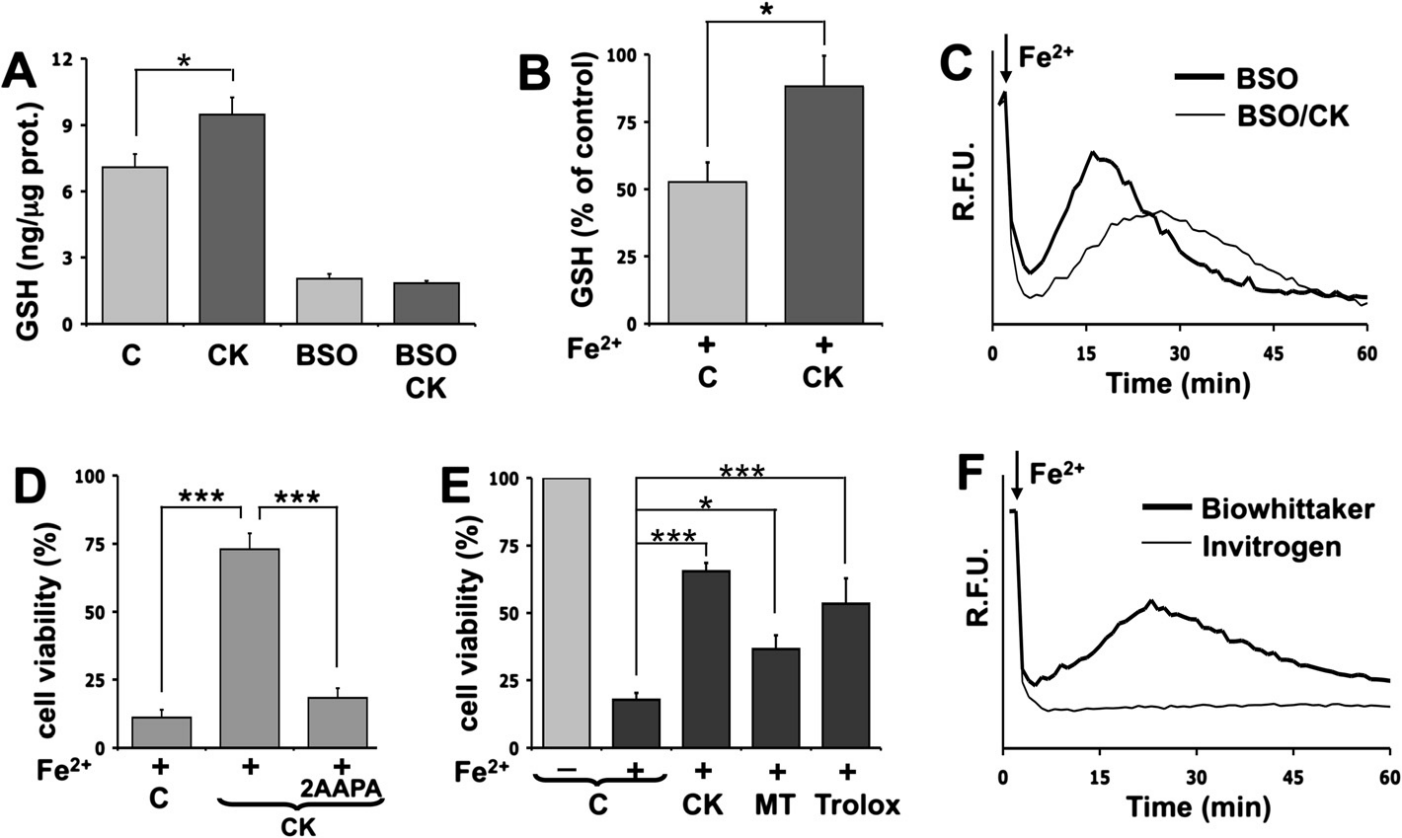
**Role of glutathione and antioxidant molecules against acute iron toxicity in astrocytes. (A)** Reduced glutathione (GSH) was measured in lysate of resting (C) or cytokine-activated (CK) astrocytes (average + SEM of three independent experiments). Statistical significance (*p < 0.05) was calculated using unpaired two-tailed Student’s *t*-test. BSO was used as a control. **(B)** GSH was measured in resting (C) or activated astrocytes (CK) in basal condition or after 30 min from iron overload. Columns represent the percent of GSH with respect to the control value of resting astrocytes (average +SEM of eight independent experiments). Statistical significance (*p < 0.05; **p < 0.01) was calculated using one-way ANOVA followed by Bonferroni. **(C)** A monolayer of resting (BSO) or cytokine-activated (BSO/CK) astrocytes was GSH depleted by BSO, loaded with fura-2 and then challenged by iron. **(D)** After 90 min from iron overload, cell viability was measured (MTT assay) on astrocytes either in resting condition (C), activated for 24 h with cytokines without (CK) or with (CK/2AAPA) 2AAPA. Each condition was expressed as the percent of viability with respect to the corresponding control without Fe^2+^ challenge. Statistical analysis in this and the following panel was performed using one-way ANOVA followed by Bonferroni (***p < 0.001). **(E)** After 90 min from acute iron overload, cell viability was measured (MTT assay) on astrocytes in resting condition (C), activated for 24 h with cytokines (CK), or pretreated for 2 h with Mito-TEMPO (MT) or Trolox (*p < 0.05; ***p < 0.001). **(F)** Astrocytes were plated in culture media containing horse serum from two different companies (BioWhittaker or Invitrogen). Cells were grown for 24 h, then loaded with fura-2 and challenged with iron overload. The traces represent the fluorescence intensity (expressed as RFU) of the astrocytes analyzed by a plate reader.

The oxidative stress, revealed by fura-2 dequenching during the iron overload protocol, was clearly the consequence of an imbalance between the ROS production and cytosolic reducing potential of astrocytes. Since a recent report showed that glutathione, the most important antioxidant molecule in eukaryotic cells, is increased upon astrocyte activation [24], we quantified the levels of this antioxidant molecule in astrocytes under our experimental conditions. We found that GSH was higher in activated than in resting astrocytes (Figure 4A), thus indicating a higher competence of activated astrocytes to maintain intracellular reducing conditions. A 24-h pre-treatment with 1 mM BSO, an inhibitor of glutathione synthesis, reduced the GSH content in both conditions and abolished any difference. Moreover, the GSH levels, after 30 min from iron overload, were significantly reduced in control conditions, but not in cytokine-treated astrocytes (Figure 4B). In light of these results, we evaluated the effects of glutathione depletion (24 h pre-treatment with 1 mM BSO) on astrocytes exposed to the iron overload protocol. In control astrocytes, both the oxidative stress and the subsequent cell death were significantly speeded up (compare the thick trace of Figure 4C with the thick gray trace of Figure 1B), while cytokine-activated astrocytes, after BSO treatment, showed a variable level of protection, ranging from complete prevention of cell death to a condition in which the damage caused by oxidative stress was delayed, but not prevented (Figure 4C, thin trace). The contribution of glutathione in the protective mechanisms observed in activated astrocytes was further confirmed by the effect of 2AAPA, an irreversible inhibitor of glutathione reductase. As expected, the decreased regeneration of GSH by treatment with 2AAPA made astrocytes susceptible to oxidative stress even in their activated state (Figure 4D). This confirms the contribution of glutathione in the protective mechanism of activated astrocytes. We also compared the protection competence acquired upon activation with the effects of exogenously administered ROS scavengers. Among the various molecules tested, we obtained significant results with Mito-TEMPO and Trolox. The first is a mitochondrial superoxide scavenger, previously shown to prevent iron-induced mitochondrial alterations in neurons [17], while the second is a water-soluble derivative of vitamin E that protects against lipid peroxidation. An analysis with the MTT assay shows that both scavengers reduce the astrocytic death caused by iron overload, but are less effective than pretreatment with IL-1β and TNFα (Figure 4E). As a note, sera from different companies and lots gave various degrees of astrocyte protection against oxidative stress. Figure 4F shows the protective effect exerted on resting astrocytes by a serum from Invitrogen compared with the one used throughout this study. A similar protective effect was also observed by using a neuronal culture medium supplemented by B27, a mix of neuroprotective and neurotrophic molecules that includes antioxidants (data not shown).

### Mechanisms involved in the protective phenotype of activated astrocytes

**Figure 5:**
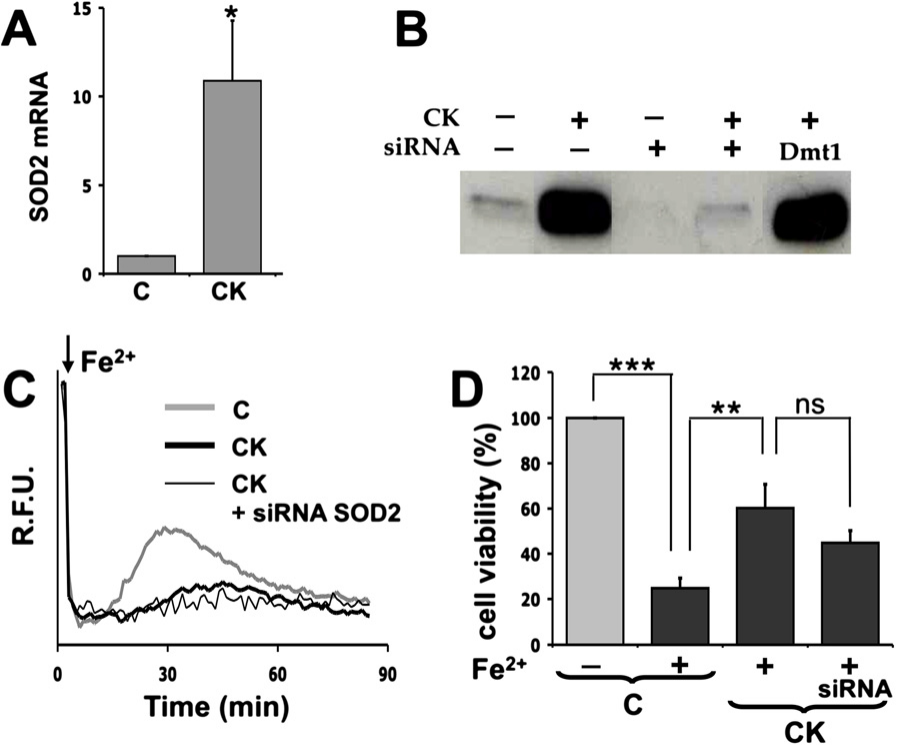
**SOD2 is not involved in cytokine-mediated protection of astrocytes. (A)** The expression of SOD2 was analyzed in resting (C) and cytokine-activated (CK) astrocytes by RT-qPCR. Statistical analysis was calculated by using paired two-tailed Student’s *t*-test. (*p < 0.05). **(B)** Resting astrocytes were incubated with lipofectamine alone (controls, for this and the following experiments), or transfected for 90 min with either a SOD2-specific siRNA or a control siRNA (against the Dmt1 transcript). After at least 6 h from transfection, astrocytes were treated with cytokines to induce activation. Efficiency of silencing was assessed by Western blot analysis. **(C)** Acute iron overload was performed on fura-2 loaded monolayers of either resting (C) or activated (CK) astrocytes with or without SOD2 silencing. Oxidative stress (recovery of fura-2 signal) and cell death (dye loss) were monitored by a fluorescence plate reader. **(D)** Ninety minutes after iron overload, cell viability (MTT assay) was measured on resting astrocytes (C) or activated (CK) astrocytes, with or without SOD2 silencing. Statistical analysis was performed by using one-way ANOVA followed by Bonferroni post hoc test. (**p < 0.01; ***p < 0.001).

In order to further investigate the mechanisms responsible for the protective phenotype of activated astrocytes, we evaluated the effects of the treatment with cytokines on the expression of proteins involved in cellular defenses against oxidative stress. We found no changes in the expression of the following proteins: catalase, SOD1, and peroxiredoxin 1, 3, 5 and 6, by Western blot analysis; in frataxin mRNA levels, by RT-qPCR; in ferritin H/L chain amount, by enzyme-linked immunosorbent assay (data not shown). On the contrary, the expression of the SOD2 protein, responsible for superoxide detoxification at the mitochondrial level, was strongly induced in activated astrocytes, and this upregulation was almost completely prevented by transfection with specific siRNAs (Figure 5A and B). Therefore, we evaluated whether the increased levels of SOD2 are responsible for the protective phenotype observed in activated astrocytes. When SOD2 induction was silenced by siRNA transfection, we did not observe a significant weakening of the protective effect produced by the two proinflammatory cytokine treatments against iron overload toxicity in either the fura-2-based assay (Figure 5C) or the MTT assay (Figure 5D).

**Figure 6:**
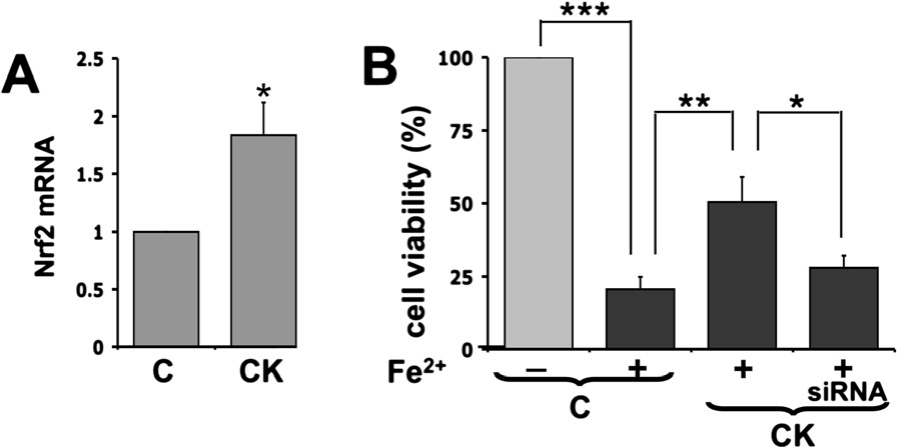
**Downregulation of Nrf2 impairs protection of astrocytes against iron overload. (A)** The expression of Nrf2 was analyzed by RT-qPCR in resting (C) and cytokine-activated (CK) astrocytes. Statistical analysis was calculated by using paired two-tailed Student’s *t*-test. (*p < 0.05). **(B)** Resting astrocytes were incubated with lipofectamine alone or transfected with a mix of two Nrf2-specific siRNAs for 90 min. After at least 6 h from transfection, astrocytes were treated with proinflammatory cytokines to induce activation. Twenty-four hours later, an acute iron overload protocol was performed, and after 90 min cell viability (MTT assay) was measured on resting (C) or activated astrocytes (CK) subjected or not to Nrf2 silencing. Statistical analysis was performed by using one-way ANOVA followed by Bonferroni post hoc test. (*p < 0.05; **p < 0.01; ***p < 0.001).

In parallel experiments we evaluated the protective response of treatments able to increase GSH via inductions of genes that are under control of the transcription factor Nrf2 [25]. As expected, chronic treatments with tert-butylhydroquinone and dopamine also produced a protective response in astrocytes (see Additional file 1: Figure S1). Since expression and activation of Nrf2 in astrocytes can modulate the expression of detoxifying enzymes involved in the oxidative stress response [26], we first measured Nrf2 levels by RT-qPCR in resting and activated astrocytes. Nrf2 expression increased upon pre-treatment with IL-1β and TNFα (Figure 6A), and this effect was reverted by transfection with specific siRNAs (data not shown). Interestingly, Nrf2 silencing reduced the protective effect of the proinflammatory cytokines, as shown by MTT vitality assay (Figure 6B), suggesting a possible role for this transcription factor in astrocyte resistance to oxidative stress.

In order to further evaluate the contribution of Nrf2 in the astrocytic defense against oxidative stress and to identify the molecular mechanisms accounting for the activation-mediated protective phenotype, we performed a wide gene expression profiling analysis in resting and activated astrocytes (not exposed to iron overload) using the Illumina RatRef chip technology. Total RNA from resting or activated astrocytes was analyzed for the expression of the ~22,000 genes available on the chip. By comparing cytokine-activated and resting astrocytes, we identified 354 genes with a >two-fold increased expression, among which 170 genes had a >3-fold increased expression; in addition, we found 253 genes with a >50% reduced expression, among which 60 genes had a >66% reduced expression. Since the phenotype obtained by exposure to the two proinflammatory cytokines is similar to that obtained by MCM[+] (Figure 3; see also [23]), we also performed the gene expression profiling on astrocytes exposed to MCM[+], obtaining similar results. In Additional file 2: Table S1, we list the genes that we found to be up- or downregulated, with a minimum of a two-fold change, after exposure to IL-1β and TNFα (CK) or MCM[+].

**Figure 7:**
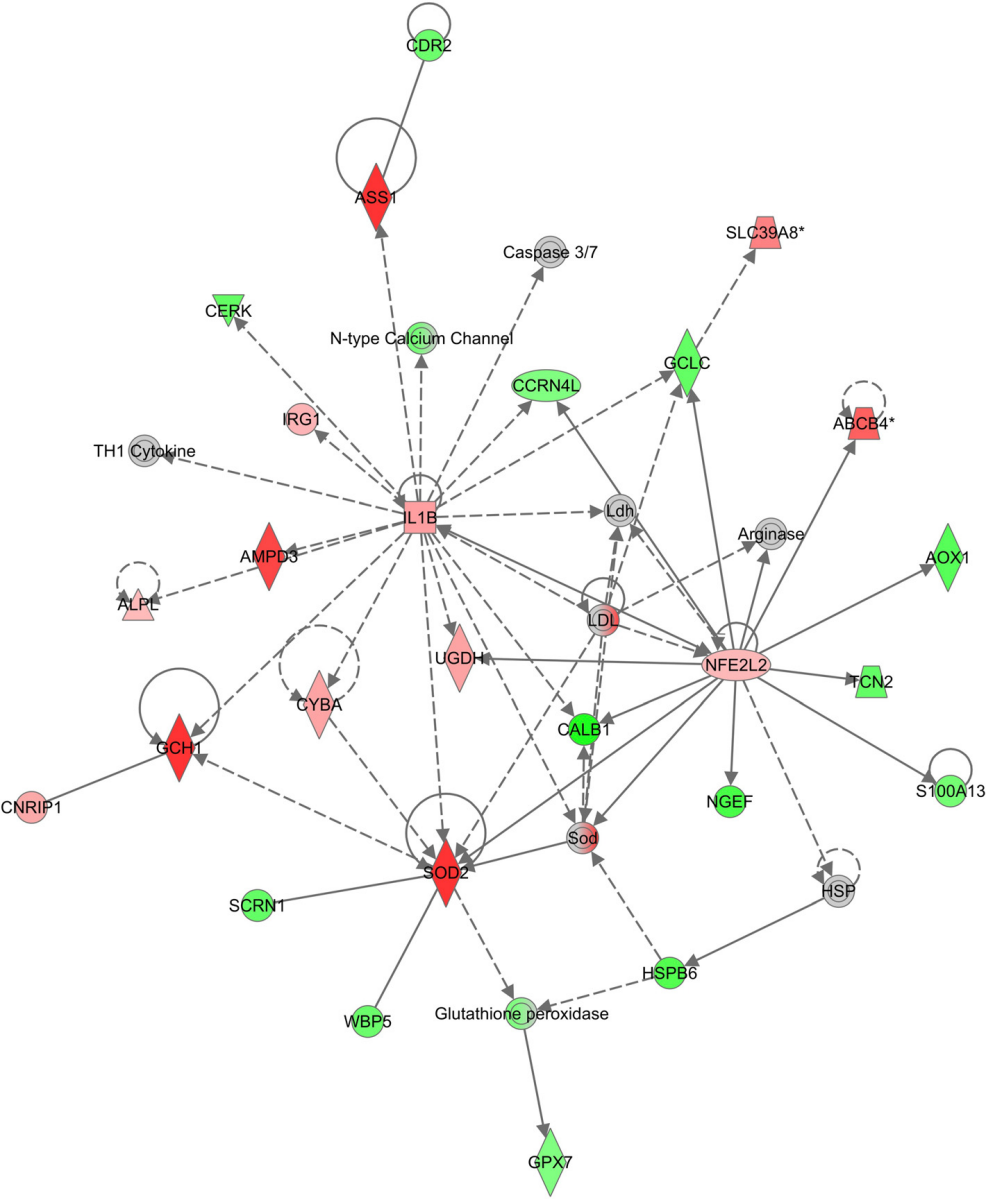
**Transcriptomic analysis of activated astrocytes reveals a modulation of genes involved in both inflammatory and the antioxidant responses.** RNA was extracted from resting astrocytes or astrocytes activated for 24 h with 10 ng/ml IL-1β and 30 ng/ml TNFα. Gene expression variations were analyzed by the Ingenuity software and graphically represented (upregulated in red and downregulated in green) as interconnections into a network.

Next, we performed a network analysis with the Ingenuity web software on the up- and downregulated molecules in activated astrocytes. The network related to oxidative stress (Figure 7) was the second best ranked (*p* < 10^-37^) and included Nrf2 (NFE2L2 in the figure), whose mRNA expression, upregulated in activated astrocytes, was linked to an increased expression of molecules involved in the inflammatory pathways, such as IL-1β, a common hallmark of astrocyte activation. Many other molecules and pathways emerged as part of this network, and the modulation of some of them was in line with our experimental evidence (i.e., SOD2, glutathione peroxidase). This bioinformatic analysis was then extended to examine first the genes identified by Röhl and colleagues [23], then to the Nrf2 pathway and, eventually, to the whole field of oxidative stress response genes.

In Table 1, the expression of the genes identified by Röhl and colleagues [23] after astrocyte activation is represented as the percentage of variation with respect to the value obtained in resting condition. Our data appear to be consistent with those obtained by Röhl and colleagues [23], and no major differences emerged between the CK (IL-1β + TNFα) and MCM[+] treatments, apart from the fact that the changes in gene expression appear to be more pronounced in our conditions of acute stimulation (24 h) than after the longer incubation time (7 days) employed by Röhl and colleagues [23].

**Table 1:** mRNA expression of selected genes under activation conditions

**Gene**	**2CK vs. C**	**MCM[+] vs. C**	**MCM[+] vs. C (Röhl et al., 2008)**
Rbp1	2,300	1,697	490
Sod2	1,511	2,322	2,200
Ass	1,422	384	135
Txnrd1	173	133	145
Gss	141	126	93
Prdx5	137	241	120
Prdx6	136	105	63
Cat	132	86	63
Gstp1	123	111	220
Hsbp1	119	92	45
Sod1	110	93	96
Mt1a	106	475	67
Nme1	104	91	80
Gstm3	102	92	62
G6pdx	101	91	100
Nme2	94	104	90
Gsr	90	104	135
Gstm1	90	116	88
Gpx1	81	65	60
Gstm2	75	120	120
Gclc	53	42	73

RNA was extracted from resting astrocytes (C, control) or astrocytes activated for 24 h with either proinflammatory cytokines (CK: 10 ng/ml IL-1β and 30 ng/ml TNFα) or conditioned medium from LPS-activated microglia (MCM[+]). The gene expression levels were determined through the Illumina BeadArray technology using RatRef-12 Expression Beadchips. The values corresponding to each gene expressed by activated astrocytes represent the percent with respect to the values of resting astrocytes. Values of the changes in gene expression relative to activated astrocytes in Röhl’s work were extrapolated from the analysis shown in the corresponding paper [23].

**Figure 8:**
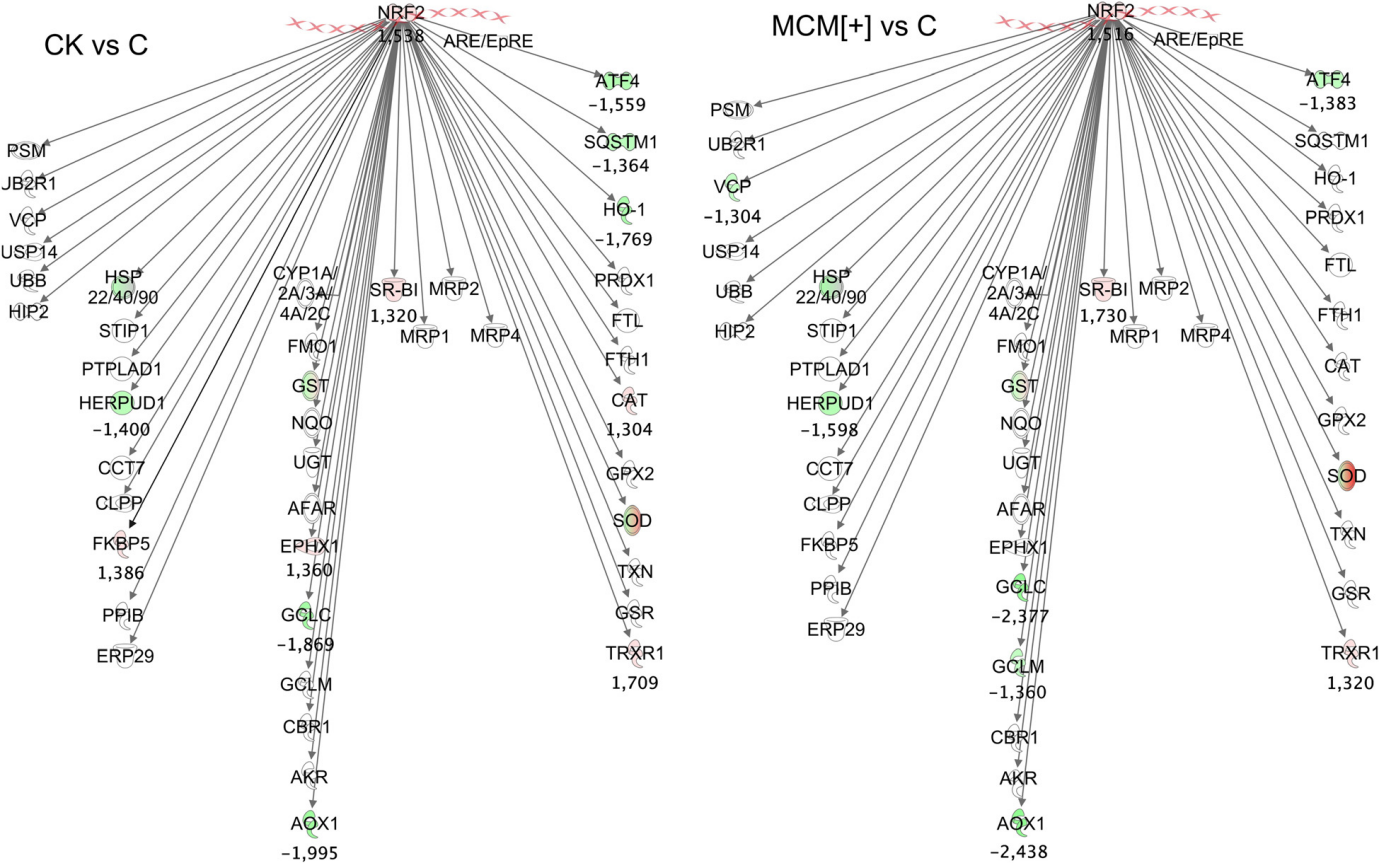
**The Nrf2 pathway is not activated after proinflammatory stimulation of astrocytes.** The canonical pathway of the Nrf2 transcription factor was analyzed with Ingenuity software. Left panel shows downregulated (green) and upregulated (red) genes in cytokine-activated astrocytes; right panel shows the same genes in MCM[+]-activated astrocytes.

In light of the importance of the astrocytic Nrf2 pathway in the protection of neurons from a wide array of insults [27], we then analyzed the transcriptomic data with the Ingenuity software to evaluate the level of activation of the Nrf2 pathway in activated astrocytes. Figure 8 illustrates the genes that are under the control of Nrf2 and that underwent downregulation (green) or upregulation (red), with respect to control expression measured in resting astrocytes, after 24 h of activation with either CK or MCM[+]. Although the transcriptomic data confirm the moderate upregulation of Nrf2 observed by RT-qPCR (see Figure 6A), the Nrf2 pathway does not seem to be responsible for the protection of activated astrocytes since the majority of the genes that are transcriptionally activated by Nrf2 remained unchanged or showed a slight tendency to decrease. Therefore, we can assume that the additional Nrf2 expressed in activated astrocytes is bound to its repressor Keap1.

**Figure 9:**
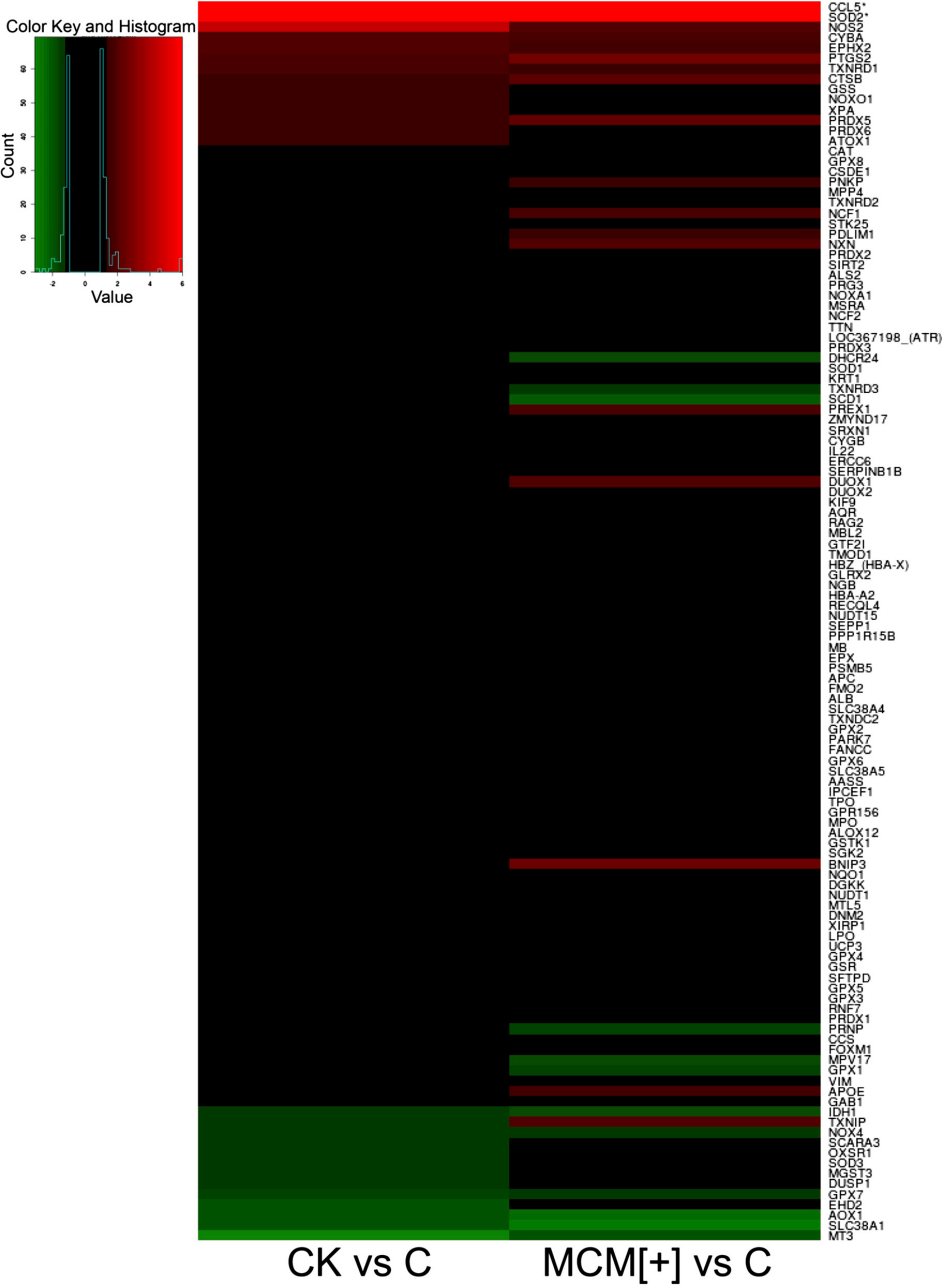
**Heat map of up- and downregulated genes after activation with CK and MCM[+].** Upregulated (red) and downregulated (green) genes are listed in descending order according to the CK stimulation (left column). The same genes are also analyzed for MCM[+] stimulation (right column).

Finally, we extended our analysis to a list of genes that are not limited to those regulated by the Nrf2 transcription factor but are commonly present in the PCR array kits designed for the study of the oxidative stress response (see, e.g., the SABioscience PCR array kit). We used a threshold that also highlights subtle changes in expression. Figure 9 shows the heat maps of upregulation (red) and downregulation (green) of these selected genes: on the left, the variation of gene expression upon CK treatment is displayed in descending order; on the right, the same genes are shown after MCM[+] treatment. In general, there were no major changes in the mRNA expression of glutathione peroxidases and peroxiredoxins, with the exception of PRXD5, which was significantly upregulated with MCM[+] treatment (see Additional file 2: Table S1). Of note, TXNRD1 (thioredoxin reductase, involved in the regeneration of peroxiredoxins) appeared slightly upregulated. Looking at the other genes that are changed with both treatments, it is possible to note the strong upregulation of the chemokine CCL5, considered an internal control of astrocyte activation, as well as SOD2 and nitric oxide synthase 2 (NOS2), as expected. Of potential interest were the upregulation of epoxide hydrolase 2 (EPHX2) and the downregulation of aldehyde oxidase 1 (AOX1), two enzymes that might play an important role in radical species detoxification and generation, respectively.

## Discussion

In this work, we evaluate the capability of astrocytes to cope with the oxidative stress induced by moderate and acute intracellular Fe^2+^ accumulation promoted by an iron ionophore. The reason behind this is that iron dysregulation and accumulation are common features of both ageing and neurodegenerative disorders and that Fe^2+^ favors the production of the highly toxic hydroxyl radicals by reacting with the H_2_O_2_ (within the framework of the self-regenerative Fenton reaction) that is physiologically produced during cellular metabolism. Under our experimental conditions, astrocytes appeared to stand the iron overload for variable periods of time, most likely up to exhaustion of their antioxidant potential. Afterward, an acute intracellular burst of oxidative stress was observed, and this is expected to reflect the reaction of Fe^2+^ with the intracellular H_2_O_2_, with consequent lipid peroxidation, loss of membrane integrity and, as a final event, cell death. The whole process occurred within minutes and appeared to depend not only on the cellular state, but also on the culture conditions. In fact, the presence of antioxidants in the culture medium was able to delay the entire process, including cell death.

Based on the work of Röhl and colleagues [23], in which astrocytes pretreated with conditioned medium from LPS-activated microglia (MCM[+]) and were found to be resistant to extracellular H_2_O_2_ administration, we considered the possibility that activated astrocytes might also be protected against the oxidative stress mediated by intracellular iron loading. In fact, the recently reported increase in iron import observed in activated astrocytes [28] could trigger oxidative stress if specific protective mechanisms are not induced.

The conversion of astrocytes from the resting to reactive state is well described in animal models of acute CNS injury and neurodegeneration [29]. However, under in vivo conditions, it is difficult to identify the causative mechanisms of the astrogliosis process, since they result from the complex integration of the responses of the various cell types present in the CNS. We reproduced the process of reactive astrogliosis by exposing astrocytes to IL-1β and TNFα, the two main cytokines released from activated microglia [30], as well as to MCM[+], a condition that is expected to better mimic the in vivo situation since LPS induces the release of a wide spectrum of proinflammatory molecules from microglia [31]. From our data, it is clear that TNFα and IL-1β are able to reproduce, in broad outline, the more complex scenario that was supposed to result from the mix of factors released by LPS-activated microglia, and, in both models, activated astrocytes acquired a protective competence not only towards H_2_O_2_, but also iron overload, with prevention of both oxidative stress and cell death. More intriguing was the analysis of the determinants of this protective phenotype. We employed a transcriptomic approach with the aim to investigate the changes in a wide panel of genes involved in the oxidative stress response. First of all, the transcriptomic analysis rules out a possible reinforcement of the control of iron detoxification, since the analysis of the transcripts did not show an increased expression of proteins involved in iron extrusion and storage. The only clear evidence from both the biochemical and transcriptomic analysis was a great increase in SOD2 expression upon activation. This draws attention to the mitochondrial enzymes that, in astrocytes exposed to inflammatory cytokines, are expected to have a pivotal role. Indeed, the dysfunction of these organelles is generally considered one of the primary causes of cell death in neurodegenerative diseases [32], a view that finds confirmation in the high sensitivity of mitochondria to ROS injury. Many antioxidant therapies have been proposed to protect mitochondria, and thus to prevent neuronal damage; however, this therapeutic strategy has turned out to be too simplistic, given the complexity of mitochondrial ROS metabolism. In fact, the process of astrocyte activation is expected to increase the mitochondrial resistance against oxidative stress by acting on a multiplicity of targets, for instance, by controlling the synthesis of SOD2, mitochondrial peroxiredoxins and glutathione. Within this complex framework, however, it is difficult to put all of them into perspective. If we consider the role SOD2 expression can play in the presence of iron overload, we can infer that removal of superoxide from mitochondria limits the self-regeneration (Haber-Weiss reaction) of the substrate for the Fenton reaction, as this molecule is able to reduce Fe^3+^ to the reactive Fe^2+^. However, superoxide dismutation also causes an elevation of H_2_O_2_, a condition that is extremely harmful in the presence of Fe^2+^ since it leads to the production of the hydroxyl radicals. Activated astrocytes appear to better deal with this problem, since they contain higher levels of not only SOD2, but also reduced glutathione with respect to resting astrocytes. Therefore, activated astrocytes appear to neutralize the harmful potential of iron by detoxifying mitochondria from superoxide as well as H_2_O_2_. Within this general view, the involvement of SOD2 appears to be less critical than that of glutathione. In fact, silencing of SOD2 did not revert the protective phenotype, while changes in the level of glutathione significantly affected the protective competence of astrocytes, suggesting that H_2_O_2_ detoxification is more critical than the disposal of excess superoxide.

Astrocytes are known to respond to oxidative stress with changes in gene expression, and the transcription factor Nrf2 was reported to play a central role in raising astrocytic antioxidant defenses [33]. In particular, a new mode of action of natural antioxidant compounds has recently emerged in which the induction of the Nrf2 pathway promotes the transcription of downstream genes involved in the protection against both oxidative stress—among others, glutathione—and inflammation [34]. However, when we exposed astrocytes to proinflammatory conditions, the Nrf2 pathway did not appear to be activated. It should be noted in this respect that the Nrf2 response against oxidative stress is activated mainly by modulation of the interaction of Nrf2 with Keap1, i.e., the repressor of its translocation to the nucleus, and not by the simple upregulation of the Nrf2 gene [35]. Overall, although activated astrocytes are reported to show a moderate upregulation of Nrf2 expression, the expression of the Nrf2 target genes was almost unaffected in our experimental conditions, leading us to conclude that the Nrf2 pathway does not contribute to the protective phenotype by increasing glutathione levels. In any event, it is clear that even if the glutathione level is important against oxidative stress, it cannot fully account for the differences observed between quiescent and activated astrocytes. In fact, even when depleted of this antioxidant molecule, activated astrocytes were more resistant to iron-mediated toxicity and cell death, thereby suggesting that other molecules potentiate the defense system. In addition to glutathione peroxidases and peroxiredoxins, recognized as key enzymes for H_2_O_2_ detoxification in astrocytes [36,37], our transcriptomic approach revealed new potential candidates for oxidative stress control. Of particular interest are TXNRD1, involved in the peroxiredoxin-mediated detoxification of H_2_O_2_ via regeneration of thioredoxins [38], and EPHX2, a hydrolase that might help to detoxify reactive epoxydes formed during oxidative stress [39]. Moreover, a downregulation of enzymes that produce H_2_O_2_, such as the aldehyde oxidase 1, which was identified in our analysis, might also contribute to decreasing the oxidative load of astrocytes. Further work is required to better define the role of these genes, and, in particular, it must be taken into account that the resistance of activated astrocytes might rely not only on changes in the expression of specific mRNAs, but also on changes in the activity of enzymes, an issue that could not be addressed by a simple transcriptomic analysis.

## Conclusions

In conclusion, the biochemical and transcriptomic analysis of astrocytes exposed to cytokines (IL-1β and TNFα) or MCM[+] suggests a complex protective mechanism that does not involve the Nrf2 pathway and might see the convergence and the synergy between several genes. The relative contribution of each of these genes has to be considered in a wider context, with particular attention to the molecules that might activate non-cell autonomous mechanisms of neuronal protection.

## Abbreviations

CNS: Central nervous system; ROS: Reactive oxygen species; CM-H2DCFDA: 5-(and-6)-chloromethyl-2’,7’-dichlorodihydrofluorescein diacetate, acetyl ester; TMRM: Tetramethyl rhodamine methyl ester; BSO: L-Buthionine-sulfoximine; GSH: Glutathione; IL-1beta: Interleukin-1beta; TNF-alpha: Tumor necrosis factor alpha; LPS: Lipopolysaccharide; MTT: 3-(4,5-Dimethylthiazol-2-yl)-2,5-diphenyltetrazolium bromide; SOD2: Mitochondrial superoxide dismutase; Nrf2/NFE2L2: Nuclear factor (erythroid-derived 2)-like 2 gene; RT: Reverse transcription; qPCR: Quantitative polymerase chain reaction; ANOVA: Analysis of variance; CK: 10 ng/ml IL-1β + 30 ng/ml TNFα; MCM[−]: Quiescent microglia conditioned medium; MCM[+]: LPS-activated microglia conditioned medium; TXNRD1: Thioredoxin reductase; NOS2: Nitric oxide synthase 2; EPHX2: Epoxide hydrolase 2; AOX1: Aldehyde oxidase 1.

## Competing interests

The authors declare that they have no competing interests.

## Authors’ contributions

RM, IP, AC and IV prepared the cells and performed the experiments. GG and FMB performed the Illumina transcriptomic analysis. RM, FC and DZ analyzed the data. RM, FC, FG and DZ conceived and designed the experimental plan and wrote the manuscript. All authors have read and approved the final version of the manuscript.

## Supplementary Material

Additional file 1: Figure S1 Chronic treatment with tert-butylhydroquinone or dopamine protects astrocytes from acute iron overload. Cell viability (MTT assay) was measured after iron overload (Fe^2+^) in resting astrocytes (C) and in astrocytes pretreated for 16 h with either 50 μM tert-buthylhydroquinone (tBHQ) or 100 μM dopamine (DA, in the presence or absence of 1 mM BSO). Click here for file

Additional file 2: Table S1 List of genes that are up- or downregulated at least two fold upon CK or MCM[+] treatment. Click here for file
