# Supplementary figures and images for: Astrocytes acquire resistance to iron-dependent oxidative stress upon proinflammatory activation
Source: J Neuroinflammation. 2013 Oct 28;10:130. doi: 10.1186/1742-2094-10-130 (PMC3874684; doi:10.1186/1742-2094-10-130)

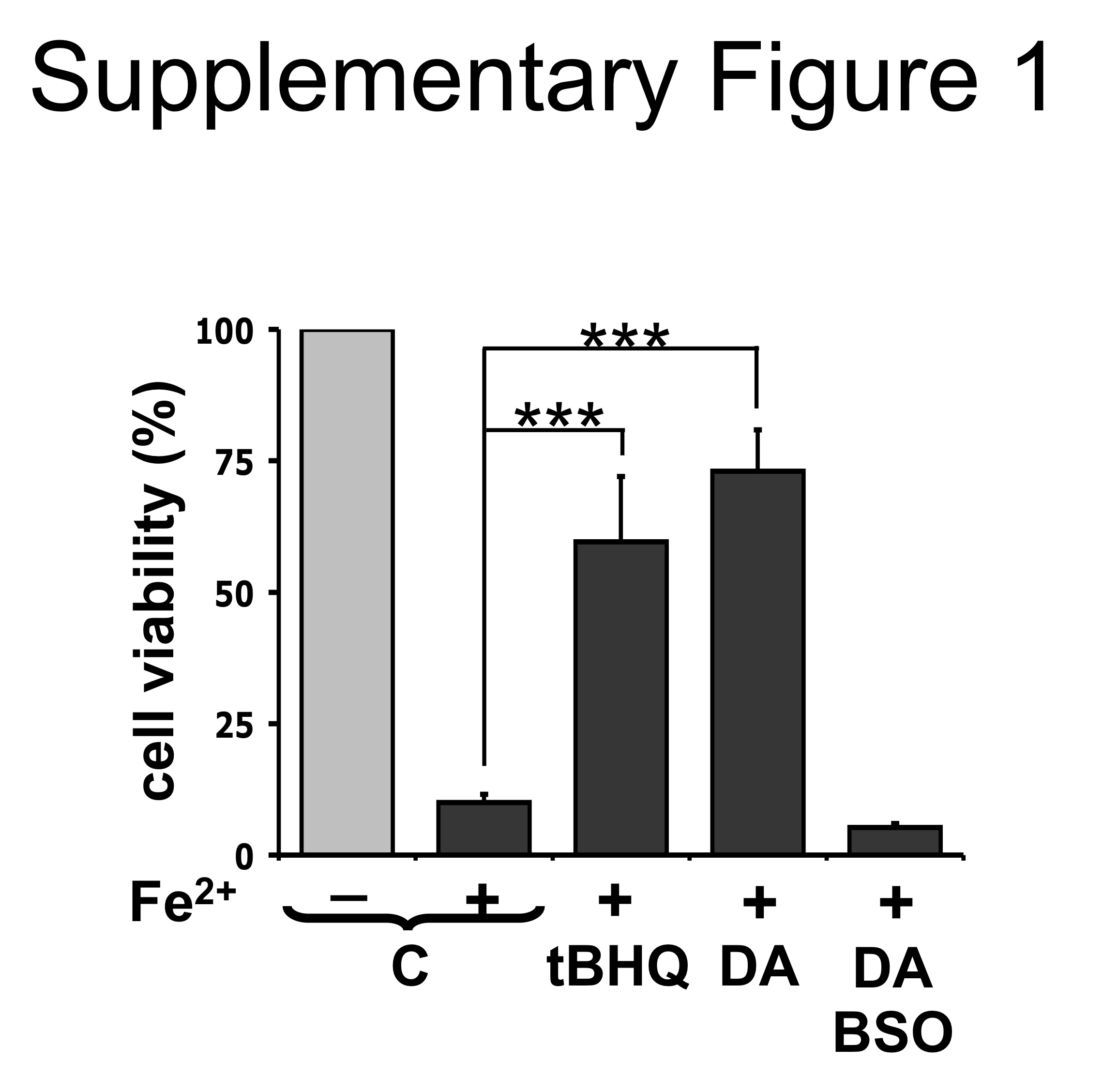

Supplement: Additional file 1: Figure S1 — Chronic treatment with tert-butylhydroquinone or dopamine protects astrocytes from acute iron overload. Cell viability (MTT assay) was measured after iron overload (Fe2+) in resting astrocytes (C) and in astrocytes pretreated for 16 h with either 50 μM tert-buthylhydroquinone (tBHQ) or 100 μM dopamine (DA, in the presence or absence of 1 mM BSO). [file 1742-2094-10-130-S1.jpeg]
