# Supplementary material for: Astrocytes acquire resistance to iron-dependent oxidative stress upon proinflammatory activation
Source: J Neuroinflammation. 2013 Oct 28;10:130. doi: 10.1186/1742-2094-10-130 (PMC3874684; doi:10.1186/1742-2094-10-130)
Supplement: Additional file 2: Table S1 — List of genes that are up- or downregulated at least two fold upon CK or MCM[+] treatment. [file 1742-2094-10-130-S2.pdf]

Supplementary Table 1

GENE	% CK vs C
Ccl5 (RANTES)	39388
Mmp3	10820
Cd83_predicted	10720
Cxcl5	10296
Ccl20	9363
Enpp2	6996
Cfb	6072
Lcn2	6058
Cxcl11	5169
LOC360228	4906
Pla2g2a	4752
Cxcl2	3115
Cx3cl1	2856
LOC497841	2699
Rbp1	2300
Ccl7 (MCP3)	2018
Ccl19_predicted	1793
Chi3l1	1627
Sod2	1511
Tlr2	1494
Mx1	1484
Slpi	1473
Vcam1	1426
Asgr1	1426
Ass	1422
Gbp2	1376
Ubd	1290
Ccl2 (MCP1)	1278
Hsd11b1	1243
Cxcl16	1220
Ptges	1214
Cxcl10 (IP10)	1203
Birc3	1040
Cyp7b1	1014
LOC311984	984
Cxcl13	937
RT1-S3	914
H2-Ea	903
Rtp4_predicted	870
Lincr	859
RGD1564528_predicted	830
Cxcl1 (GRO1)	761
LOC363060	759
Gbp5_predicted	727
Scn4b	704
Jak2	694
Casp1	689
Gch1	673
LOC294942	673
Cxcl12	673
Cd302	667
Tnfrsf11b	661
Cd69	659

GENE	% MCM[+] vs C
Cfb	25891
Cxcl5	14091
Ccl20	9745
Slpi	8253
Lcn2	7882
Chi3l1	7565
Cxcl11	4800
Cd83_predicted	3067
Ccl5 (RANTES)	2734
Pf4	2369
Enpp2	2332
Sod2	2322
Pla2g2a	2234
Orm1	2216
Mgp	2155
Ccl7 (MCP3)	2107
Ptges	2096
LOC497841	2047
Cxcl2	2000
Serping1	1997
Asgr1	1744
Rbp1	1697
Slc16a3	1614
Rtp4_predicted	1588
Mx1	1576
Mmp3	1539
LOC360228	1348
Serpina3n	1226
Sfrp2	1212
Tlr2	1173
RT1-S3	1170
Ccl2 (MCP1)	1169
Cyp7b1	1119
Cd302	1085
Birc3	1058
Oasl2	1039
isg12(b)	1020
Vcam1	1016
Tf	988
Ccl6	962
Il1b	949
Cxcl12	946
LOC498276	932
Ifit1	920
Cxadr	890
Irf7	877
Slc39a8_predicted	830
Cxcl9	781
Ccl19_predicted	770
Slc39a8	768
Gbp2	754
Gch1	753
Cx3cl1	735

Pde4b	657
RGD1560020_predicted	655
Ifngr1	635
Oasl2	623
Upp1	618
Mgp	604
Madcam1	590
Tnip1_predicted	575
Nfkb2	573
RGD1561113_predicted	571
Irf1	570
Msln	567
Slc1a1	563
Nos2 (iNOS)	560
Cxcl9	550
Icam1	550
Tf	550
Has2	546
Psmb9	534
RT1-A2	533
Irf7	533
Mmp9	529
Sh2b2	504
Abcb1b	501
Nfkbia	493
Tnnt2	491
RGD1563437_predicted	491
Slc13a3	490
Fgr	487
Map3k8	485
Cdc42ep2	484
Emr1	480
Psmb8	473
RGD1563354_predicted	468
Csf1 (M-CSF)	467
LOC310968	464
RT1-149	464
LOC501092	462
Serping1	457
LOC289918	456
RT1-A3	452
Csf2	450
Rgs7	450
LOC288515	444
RT1-CE15	444
Nrep	439
Cyp1b1	438
Hdc	437
Slc11a2 (DMT1)	435
Gypc	434
Nfkbie	433
Ibrdc3_predicted	432
Plxnd1_predicted	432
Ifit1	430
Ripk2	427
C1s	425

LOC289918	733
Rarres2	728
RGD1564528_predicted	726
Cxcl16	723
Cxcl1 (GRO1)	705
LOC363060	694
Jak2	690
Cebpb	665
Pde4b	656
Upp1	653
Ccl3 (MIP1alpha)	649
Msln	649
Pdgfra	648
LOC311984	629
C2	628
LOC294942	598
RGD1561113_predicted	596
Rnf125_predicted	577
Ak3l1	577
RGD1310093_predicted	576
Cxcl13	571
Mx2	566
Cxcl10 (IP10)	565
Ifi47	563
LOC299279	560
Steap1_predicted	559
Hpgd	558
Fgr	549
Ngfb_mapped	548
RGD1564259_predicted	543
RT1-CE7	539
Ifi27l	536
Rnd1	515
Slc1a1	509
C1s	508
LOC498277	507
RT1-A2	505
Oas1a	504
Il1a	489
Cipar1	482
Col14a1_predicted	479
Mt1a	475
Ccl4	472
LOC497811	469
Hsd11b1	468
Gja5	463
Casp1	461
Ube2l6	453
Ifitm1_predicted	451
Emilin1_predicted	448
Cyp1b1	447
Oas1i	446
Nfkbia	444
Mmp9	440
LOC498279	436
C1qc	435

Ifi47	424
Camk1g	418
RT1-CE7	416
Sh3gl3	412
Ampd3	411
Clu (clusterin)	411
LOC497812	406
Stap2	398
RT1-M6-2	392
Sept4 (38963)	389
Pla1a	388
Zc3h12a_predicted	388
Htr7	387
Apol3_predicted	383
Cebpb	383
Ube2l6	378
Lgals3bp	377
RT1-A1	377
Psmb10	376
Gpr176	371
Psme2	371
Klf4	367
LOC299339	365
Birc2	361
Cdc42ep5_predicted	361
Ua20	356
Smoc2_predicted	354
Ngfb_mapped	352
Slc15a3	351
Xcl1	348
Pde9a	348
Msc_predicted	346
Irf6_predicted	343
RGD1563091_predicted	332
LOC498371	325
Ppap2b	325
Zmynd15_predicted	322
Gad1	321
Irgm	320
Tifa	320
LOC501245	317
Nfkbib	317
LOC689135	317
Herc6	317
Pdgfra	316
H2-M10.6	315
RGD1565710_predicted	314
Cd74	313
Rnf125_predicted	312
Tnfaip8_predicted	312
Bckdhb	310
LOC691575	310
Slc6a6	309
Ifi27l	304
Olr472	304
Agtr1a	302

Rasd1	433
Cpxm1_predicted	428
Lgals3bp	425
Tnip1_predicted	415
RT1-CE15	412
LOC497812	412
Icam1	409
RT1-M6-2	409
RGD1562052_predicted	409
Ppap2b	404
Lincr	402
Map3k8	402
Sept4 (38963)	400
Lum	400
Bst1	398
Clu (clusterin)	396
Nfkb2	396
LOC310968	394
Gbp5_predicted	392
H2-Ea	388
Serpine1	386
Oasl	385
Pde9a	385
Ass	384
Birc2	383
Sst	382
Psmb8	381
Ccl12_predicted	380
Nell2	374
LOC686871	373
Vgf	372
RT1-A3	370
Fam38a_predicted	370
Ctss	370
Irgm	366
Angptl4	362
RGD1563091_predicted	362
Ms4a4a_predicted	361
Nfkbiz_predicted	357
Zc3h12a_predicted	356
RT1-149	354
Bckdhb	354
Adora2a	351
Gpr88	348
Ifngr1	346
C1r	346
RGD1311559_predicted	345
Aebp1_predicted	343
Aif1 (IBA1)	341
Tnn_predicted	341
C1qb	341
Tgm2	340
Slc11a2 (DMT1)	339
Herc6	339
Timp1	338
LOC499985	338

RGD1311559_predicted	302
Orm1	302
C2	302
Asam	301
RGD1559845_predicted	301
Casp11	298
LOC363320	294
LOC691487	290
Npb	290
LOC363434	289
RT1-Ba	288
LOC497769	288
RGD1562717_predicted	287
Slc39a8_predicted	286
Ikbke_predicted	286
Hpx	285
Nfkb1	284
Pappa_predicted	283
LOC497768	282
Nov	281
RGD1560723_predicted	278
RGD1309362	278
RGD1309906	276
LOC365510	275
Hla-dmb	275
Esm1	275
Junb	275
Edn1	274
Slc6a4	274
Sp110	272
LOC501224	272
Rbp2	270
Ifngr2_predicted	269
Serpina3n	269
RGD1560819_predicted	268
Cpxm1_predicted	266
P2rx5	266
Il18bp	265
Ifi44	265
Flrt3_predicted	264
Adprtl1	264
Sfrp2	263
RGD1310093_predicted	261
Scin	261
Abcb4	261
Slc39a8	260
Crabp1_mapped	260
LOC300043	259
Tbc1d22b	259
LOC686871	258
Pnrc1	258
Oas1a	257
RGD1310490_predicted	257
Serpine1	257
Nrp1	257
Fgf13	257

Irak3_predicted	337
Ndrg1	335
Tmem176b	335
Edf1_predicted	335
Scin	334
Fcgr3	330
Lgi2_predicted	326
Runx1	326
Ifitm3	325
Pygl	324
Tnfaip8_predicted	324
LOC315106	322
LOC681872	322
Psmb9	321
Tmem106a	321
Sp110	319
Slc15a3	319
Hla-dmb	318
RGD1564163_predicted	316
Sh2b2	315
Arg1	311
Dnm1	309
Irf1	309
Osmr	308
Il1rn	306
Tapbp	306
Arhgdib	305
Mgst2_predicted	305
Mitd1	304
Pnrc1	302
Cdc42ep2	300
Pfkl	300
Nrip1_predicted	297
Pappa_predicted	297
H2-M10.6	297
S100a8	295
Scn4b	293
Sulf1	293
RGD1308126_predicted	293
LOC288515	291
Slfn3	291
Ifi44	291
Hmgn3	291
Ereg	289
RGD1562323_predicted	289
Apln	288
RGD1310490_predicted	287
Tcea3	287
Npb	286
Enpp3	286
Olr472	286
RGD1309759_predicted	285
Htr7	284
Il33	284
Junb	283
Has2	283

Stat1	257
RGD1564259_predicted	256
LOC316186	255
Csf3	255
H2-M3	255
Ikbkb	255
Prnd_predicted	255
Herc1_predicted	255
Fam38a_predicted	253
Irf5_predicted	253
Klf15	251
Bmp2	251
Rassf9	250
Irak3_predicted	249
Akr1b8	248
Psme1	248
Plk4_predicted	247
LOC499365	247
Nrip1_predicted	246
C1r	244
Fam101b	244
Oas1i	244
Il1f8_predicted	244
Steap1_predicted	242
Slc1a2	241
Stard4_predicted	241
Slco3a1	239
Nmi	238
Tmem123	238
Tmem140	238
Plat	238
Nfkbiz_predicted	237
Ccl12_predicted	236
Atp11a_predicted	236
S100a5_predicted	236
LOC500300	236
LOC501250	236
Eno2	236
Tapbp	236
Olr1744	235
Btg1	235
Slc16a3	234
Mitd1	234
isg12(b)	234
RGD1309759_predicted	233
LOC501400	233
Tap1	232
RGD1561715_predicted	232
LOC287622	232
Rarres2	231
RGD1560022_predicted	231
Col4a1	231
RGD1306494_predicted	231
Cd1d1	231
Epn3	230
Tgm2	230

Psme2	283
Ppp1r3c	282
Tgm1	282
Tifa	282
Ua20	282
Emr1	282
Mrc1_predicted	281
Ptgs2 (COX2)	281
Cesl1	280
Thbs4	279
Csf3	279
Mgst1	277
Nfkbie	277
Nfkb1	276
RGD1309906	276
Ibrdc3_predicted	275
Ch25h	275
Slc16a1	275
Cd36	275
LOC500687	274
LOC299339	272
Batf3	271
RT1-A1	270
Cd82	268
LOC689135	267
Klf15	267
Stat2	266
RGD1309362	266
Ier3	265
Rit2	265
Itga7	264
Cyp4b1	263
Ugcg	263
Gap43	261
Eno2	260
Cfd	260
Il6	258
Slc9a3r1	257
Cd68	257
Rgs7	256
Abcb4	255
Pla2g4a	255
Arg2	254
Adm	254
Abcb1b	252
Fmo5	252
RGD1561715_predicted	251
Btg1	250
March3 (38778)	250
Asam	250
Acsbg1	250
Irf9	249
Serpinb2	249
Bcl2a1d	249
Lcp1	249
Gda	249

Ptprv	230
P4ha2_predicted	229
Hmgn3	229
Itga7	229
Runx1	228
Angptl4	228
Ccrl2_predicted	227
Pkp1_predicted	226
Ralgps2	226
Ets1	225
Cd44	224
Lrrc8c	224
Lad1_predicted	224
Gpr88	224
Il1b	224
Edf1_predicted	222
Tcirg1	222
Cxadr	222
Vgf	222
LOC301044	221
Rhbg	221
Mgmt	220
Mx2	220
Cd82	220
St6gal1	220
Acsbg1	219
Tcea3	219
LOC690672	218
Svil_predicted	218
RT1-T24-1	218
Btg2	217
Slc24a6	217
Litaf	216
Lin7a	214
Cd72	214
Adrb2	213
LOC689770	213
Il1rn	213
Cib1	212
Stx6	212
LOC500687	212
Cd47	211
March3 (38778)	210
Sp140	210
Phlda2_predicted	209
LOC501399	209
Cdc42ep3_predicted	209
LOC315106	209
Es1	209
Sulf1	209
LOC500420	207
RGD1305179_predicted	207
Tcp11	207
Gadd45b	207
Gls2	207
Oasl	206

Nid2	249
Cdc42ep5_predicted	248
Gpr176	247
LOC365510	247
Bnip3	247
Apol9a	245
LOC300043	245
Abca1	243
Gypc	243
Prdx5	241
Tap1	241
Cd1d1	241
Stat3	240
Creb3l1	240
Sh3gl3	239
Adamts5	239
Hpx	239
Casp11	239
LOC305633	239
Pgf	238
Mapkapk2	236
Ampd3	236
LOC312688	236
Gpam	235
St6gal1	234
Adcy2	234
Nfkbib	233
Nppc	233
LOC297481	233
RGD1565371_predicted	233
Stat1	232
Cd14	232
Tnnt2	232
Ikbke_predicted	231
Saa4	230
Ms4a11_predicted	230
Lin7a	230
LOC497816	230
Nov	229
Mafb	229
Coro1a	229
RGD1309576_predicted	229
Tnfrsf11b	229
Akr1b8	227
Btg2	227
Anpep	227
Glrx1	227
Igtp	226
RGD1565715_predicted	226
Es1	225
Csf1 (M-CSF)	225
Cbx7	224
LOC499365	224
Zfp36l1	224
Abca2	223
RT1-N3	223

Guca2b	206
LOC500590	206
RGD1559885_predicted	205
Il33	204
Bdkrb1	203
Sema4a	203
Pvalb	203
Bst1	203
LOC363181	202
Scly	202
RGD1563599_predicted	202
Pcsk2	201
Rnd1	201
Baiap2	201
Zbp1	201
Snf1lk	201
Casp7	200
Ugdh	200
Cesl1	200
Mgst1	200
Gsdmdc1_predicted	200
Mcam	50
Cspg2	50
Tm4sf2_mapped	50
Gem_predicted	50
Phldb1	50
Igsf4c_predicted	50
Tmepai_predicted	50
Aox1	50
Cpxm2_predicted	50
RGD1311080_predicted	50
Oplah	50
LOC500297	50
RGD1310827	50
Sfxn5	50
Gfra1	50
RGD1564040_predicted	50
Iqsec3	49
Map2	49
RGD1565999_predicted	49
Akt1	49
Epb4.1l3	49
Naprt1	49
Ctsc	49
RGD1561795_predicted	49
LOC499741	49
Nuak2	49
Cav3	49
Pck2_predicted	49
Slc38a1	49
Ube2t_predicted	49
RGD1307524_predicted	49
RGD1563615_predicted	48
Zic2_predicted	48
LOC500506	48

Il1rl1	223
Il18bp	223
LOC363267	222
Ugdh	222
Adamts1	222
C4-2	220
Tcp11	220
RGD1560819_predicted	219
Cd44	218
Ptpn1	218
Fmod	218
Tyrobp	218
Msc_predicted	217
Sema4a	217
Ctsb	217
Hhex	217
RGD1563354_predicted	216
Nmi	216
H2-M3	216
Fcgr3a	215
Socs3	214
Plxnb2	214
Ahr	214
Siglec10_predicted	214
LOC499196	214
Plxnd1_predicted	213
Rela	213
RGD1560496_predicted	213
C1qa	212
Ece1	211
Herc1_predicted	211
RT1-T24-1	211
Ppap2a	211
LOC500959	211
LOC501211	210
Sirpa	210
Esm1	210
Bcl6_predicted	209
Nos2 (iNOS)	209
LOC307731	209
Lama1_predicted	207
Trim34_predicted	207
Tmem123	207
Atp11a_predicted	207
Rbp2	207
Plac8_predicted	206
Rdh10	206
Rac2	206
Laptm5	206
P4ha2_predicted	206
Lrig1_predicted	206
Ptafr	206
Ogfrl1	206
Hif1a	205
RGD1306494_predicted	205
RGD1559845_predicted	205

F2r	48
LOC361480	48
MGC72614	48
Clstn1	48
Cthrc1	48
Mfap4	48
Fgfr3	48
LOC302405	48
Trib3	48
Ddx21a_predicted	48
Actn4	48
Pcdh8	48
Uts2r	48
Lrp4	48
RGD1559565_predicted	48
Gpcr12	48
LOC497708	48
LOC497673	48
Hspb6	48
Pcp4	47
RGD1565099_predicted	47
Rhob	47
LOC497892	47
Mamdc2	47
Epha5	47
Abcb9	47
Pcdh17_predicted	47
Psph	47
Sparcl1	47
LOC498795	47
RGD1305844	47
Npy	46
RGD1311530_predicted	46
Ednrb	46
Cd59	46
Lrrc48	46
Cubn	46
Pdgfc	46
LOC361143	46
Ccdc37_predicted	46
Tnr	46
Boll_predicted	46
Pea15a	46
Emp1	46
LOC497844	46
LOC361841	46
PRKCQ	45
Col8a2_predicted	45
Ntrk3	45
Tc2n	45
Cyp4b1	45
Paqr4	45
Mdga2	45
LOC501633	45
Ankh	45
Tle3	45

Apol3_predicted	204
Duox1	204
RGD1561940_predicted	204
Ppif	203
Klf4	202
Ston2_predicted	201
Ikbkb	201
Centg2_predicted	201
P2rx5	200
Ifi204	200
Schip1	50
Cars_predicted	50
Cldn19	50
Odc1	50
Arhgap21_predicted	50
Prkag2	50
Rapgef4	50
Hsd17b7	50
Bambi	50
Cldn9	50
Abhd1	50
Gucy1b3	50
LOC361480	50
Hs3st1	50
Sc5dl	50
Plcl1	49
Abcb9	49
Ankh	49
Fat3	49
RGD1309107	49
Gpc4	49
Klhl13	49
Slc7a1	49
RGD1562701_predicted	49
Cpa2_predicted	48
Arvcf_predicted	48
RGD1562618_predicted	48
Rarres1	48
Cspg4	48
RGD1564335_predicted	48
Kcnd3	48
Bhlhb3	48
Olig1	48
LOC497806	48
Trim9	48
Hspb6	48
Tm4sf1_predicted	48
Cltb	47
Apeg3	47
Uts2r	47
LOC497892	47
Myl9_predicted	47
LOC501268	47
Fstl5_predicted	47
RGD1561795_predicted	47

Nefl	45
Ahrr	45
Chdh	45
Ezr	45
Zfp312_predicted	45
Crhbp	44
Plec1	44
Eln	44
Plcl1	44
Myl9_predicted	44
C1ql1_predicted	44
LOC499856	44
Htra3_predicted	44
Plxdc2_predicted	44
RGD1561090_predicted	44
Metrn	44
Prom1	44
Slc5a3	44
Wfdc1	44
Ascl1	43
Snurf	43
RGD1311307	43
Ngef_predicted	43
Acot1	43
Ptms	43
Timp2	43
Msn	43
Gpc4	43
RGD1308329_predicted	43
RGD1561817_predicted	43
Ephb1	43
Angptl2	42
Thrsp	42
Chac1_predicted	42
Vldlr	42
Sema7a_predicted	42
Tm4sf1_predicted	42
Asphd2	42
S100b	42
Stmn2	42
Gamt	42
Chst1	42
Asns	41
Olfm1	41
Aldoc	41
Pthr1	41
Ppp1r1b	41
Fos	41
Cltb	41
Phgdh	41
Gabrb1	40
Slco1c1	40
Dmd	40
RGD1563465_predicted	40
Atf5	40
Kcnd3	40

RGD1309930	46
Fbn2	46
Cacng4	46
Cd24	46
Iqsec3	46
Mfap4	46
MGC112883	46
Ocln	46
Eln	46
Myh11	46
Id2	46
Sdc2	46
Dhcr7	46
Folh1	46
Epb4.1l3	46
Mybl1_predicted	46
Sc4mol	45
LOC501633	45
Cubn	45
LOC498795	45
Tsc22d3	45
Zfp312_predicted	45
Ppp1r1b	45
Eno3	45
Cxxc5	45
Loxl2_predicted	45
Trib3	45
Scd1	44
Prkcb1 (PKCbeta)	44
Slco1c1	44
LOC362870	44
Tnr	44
RGD1308967_predicted	44
RGD1564008_predicted	44
Sox6_predicted	44
RGD1561090_predicted	44
Mgll	44
LOC499741	44
Ccdc37_predicted	44
Nefl	44
Nes	43
Ucp2	43
Ntrk3	43
Cd59	43
Ppp2r2b	43
Chac1_predicted	43
LOC290704	43
Gabrb1	43
Pcdh17_predicted	43
Prom1	42
Scrg1	42
Npvf	42
LOC363498	42
Scrn1	42
Map1b	42
Slc15a2	42

Myh11	40
Mlc1_predicted	40
Kcnk3	40
Slc7a3	40
LOC684490	40
Sult1a1	39
RGD1564335_predicted	39
Hspa2	39
Fstl3	39
Ckb	39
Klhdc8b	39
Atp1b1	39
Jun	39
Hspb1	38
RGD1311049_predicted	38
Tmbim1	38
Gdpd2_predicted	38
Ssg1	38
Sidt2_predicted	38
Gnao1	37
Errfi1	37
Igfbp3	37
Crip2	37
Cldn19	37
Fbln2	37
LOC316573	37
RGD1308967_predicted	36
Ddr1	36
RGD1560177_predicted	36
Boc_predicted	36
Myo16	36
MGC112790	36
Myh10	36
Akap12	36
Gpr56	36
Fbxo2	36
Ca3	36
Scn7a	36
Leprel1	35
Actg2	35
Gfap	35
Tinagl1	35
Cacng4	35
Pcbp3_predicted	35
Pmp22	34
Rgs2	34
Ctnnal1_predicted	34
Car9_predicted	34
Slc15a2	34
Map1b	33
Igsf11	33
LOC362870	33
Col11a1	33
Mgll	33
Mt3	33
Lpl	33

LOC302022	42
Gclc	42
Ptpro	42
Foxd4	42
Cpxm2_predicted	42
RGD1307150_predicted	41
RGD1564040_predicted	41
S100b	41
PRKCQ	41
Gpm6a	41
RGD1308329_predicted	41
Fbxo2	41
Aox1	41
Adamts9_predicted	40
Emp1	40
Tc2n	40
Tle3	40
Ap1s2_predicted	40
Wfdc1	40
Rpe65	40
Prkcdbp	40
Tmbim1	39
Scn3b	39
Trpm6_predicted	39
Cyp51	39
Idi1	39
Fdps	39
Hopx	39
Nrg1	39
Gulp1	39
LOC497703	39
Gnao1	39
LOC497814	39
Adra1b	39
Ncam1	39
RGD1563615_predicted	39
Gpr56	39
Slc27a3_predicted	38
Lrrc48	38
Ttyh1_predicted	38
Itga11_predicted	38
Kcnk3	38
Atp1b1	38
Ascl1	38
Crhbp	37
Lxn	37
Fbln5	37
Tpm1	36
Phgdh	36
Sult1a1	36
Ephb1	36
Myo16	35
Tinagl1	35
Hmgcs1	35
Galntl1	35
Slc38a1	34

Sez6	33
Nes	32
Chst2_predicted	32
Timp3	32
Dmn	32
Cxcr4	31
Slc39a12_predicted	31
Scrg1	31
Tpm1	31
Col12a1	31
Adamts9_predicted	30
RGD1305809_predicted	30
RGD1310174_predicted	30
Slc7a5	30
Sorl1_predicted	30
Tagln3	29
Cyp1a1	29
Cryab	29
Maob	28
Fbln5	28
Cnn1	28
Cldn9	27
Lsamp	27
LOC311772	26
Sostdc1	25
Slc6a15	25
Flnc_predicted	25
Aldh1a1	25
Fhl1	25
Tmeff2_predicted	24
Cd24	24
Tgfb3	24
Klhdc8a	24
Fgfbp1	24
Omd	24
Col6a3_predicted	24
Rpe65	24
RGD1310242	23
Gldn	23
Fez1	22
Atp1a2	21
RGD1564664_predicted	20
Gpr37l1	20
Galntl1	20
Ogn_predicted	19
Gria2 (AMPA-R)	19
Ttyh1_predicted	19
Atp1b2	19
RGD1560183_predicted	18
LOC499513	15
Calb1	12
LOC363443	11
Acta1	10

Neto2_predicted	34
LOC498228	34
Sostdc1	34
Tm7sf2	34
Igsf11	34
RGD1563465_predicted	33
Chst2_predicted	33
Timp3	33
Cyp1a1	33
RGD1311307	33
Sorl1_predicted	33
Lsamp	33
MGC112790	33
Tmepai_predicted	32
Lpl	32
Cxcr4	32
Psph	32
RGD1310242	32
Leprel1	32
Ctnnal1_predicted	31
Dmd	31
Fhl1	31
Aldh1a1	31
Asns	31
Tgfb3	31
Tagln3	30
Lcat	30
RGD1305809_predicted	29
LOC684490	29
LOC316573	29
Dmn	29
Sidt2_predicted	29
Fez1	28
LOC499856	28
Actg2	28
LOC500297	28
MGC72614	27
Atf5	27
Slc6a15	27
Col11a1	27
Klhdc8a	26
Mal	25
Fgfbp1	24
RGD1310174_predicted	24
Slc39a12_predicted	24
Igfbp3	24
LOC311772	23
Acot1	23
Cryab	22
Pcp4	22
RGD1560183_predicted	21
Gldn	20
Hmgcs2	19
Gria2 (AMPA-R)	19
Slc7a3	19
Slc7a5	18

Ca3	18
LOC499513	15
Omd	14
Ogn_predicted	13
LOC363443	11
Calb1	9
Acta1	6
